# PLGA/SF/linagliptin wound matrix-induced membrane promotes diabetic wounds healing by inhibiting macrophage pyroptosis

**DOI:** 10.1093/rb/rbag048

**Published:** 2026-05-08

**Authors:** Yizhuo Ren, Yanlong Wang, Kaixuan Cai, Xianzheng Lin, Tao Tang, Meng Yin

**Affiliations:** Department of Cardiothoracic Surgery, Shanghai Children’s Medical Center, Shanghai Jiao Tong University School of Medicine, Shanghai 200127, China; Department of Cardiothoracic Surgery, Shanghai Children’s Medical Center, Shanghai Jiao Tong University School of Medicine, Shanghai 200127, China; Department of Burns and Plastic Surgery, Shanghai Children’s Medical Center, Shanghai Jiao Tong University School of Medicine, Shanghai 200127, China; Department of Burns and Plastic Surgery, Shanghai Children’s Medical Center, Shanghai Jiao Tong University School of Medicine, Shanghai 200127, China; Department of Burns and Plastic Surgery, Shanghai Children’s Medical Center, Shanghai Jiao Tong University School of Medicine, Shanghai 200127, China; Department of Cardiothoracic Surgery, Shanghai Children’s Medical Center, Shanghai Jiao Tong University School of Medicine, Shanghai 200127, China

**Keywords:** DPP-4 inhibitor, electrospinning, chronic inflammation, skin regeneration

## Abstract

Diabetic wounds often exhibit delayed healing because of persistent inflammation, thus, remaining in the inflammatory phase. A key factor driving the delayed healing is pyroptosis, a proinflammatory form of programmed cell death which leads to the excessive release of inflammatory mediators. And macrophages are deeply involved in the entire process of wound inflammation. In this study, to regulate the pathological inflammation, we developed linagliptin-loaded hybrid PLGA [Poly(lactic-co-glycolic acid)]/SF(Silk Fibroin) membranes by electrostatic spinning. This advanced biomaterial with suitable physicochemical properties and biocompatibility is designed to provide a sustained release of linagliptin. Our *in vitro* experiments demonstrated that linagliptin effectively suppresses the macrophage pyroptosis through the noncanonical pathway, thereby reducing the secretion of key pro-inflammatory factors and improving the function of cells involved in wound healing. Furthermore, our *in vivo* experiments demonstrated that the PLGA/SF/linagliptin membrane promotes the healing of diabetic wounds and shortens the inflammatory phase of wounds, while improving skin regeneration. The collective results confirm the linagliptin’s capacity to inhibit nonclassical pyroptosis and the PLGA/SF/linagliptin wound matrix-induced membrane’s potential to promote wound healing. In conclusion, we have developed a novel wound matrix-inducing membrane with slow linagliptin release that promotes chronic wound healing by modulating macrophage pyroptosis.

## Graphical abstract

**Graphical Abstract:**
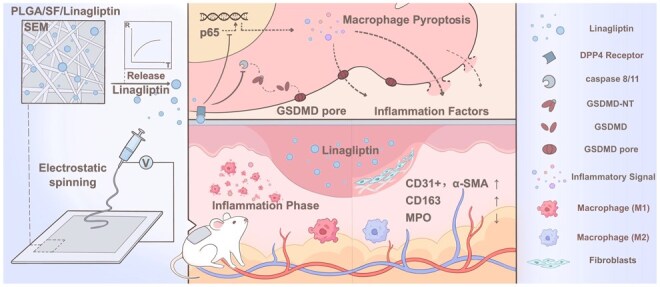

## Introduction

Diabetes places significant strain on the global healthcare system, and diabetic ulcers are one of the major complications in this group of patients. Notably, about 20% of moderate-to-severe diabetic ulcers require amputation treatment. Statistically, diabetic ulcers are the leading cause of amputation in nontraumatic patients in the USA, costing approximately $10 billion in healthcare expenditures annually [1]. Physiologically, normal wound healing involves hemostasis, inflammation, cell proliferation and extracellular mesenchymal remodeling, which require coordination between different types of cells [2]. In contrast, chronic hyperglycemia leads to excessive glycation of functional proteins in tissues in diabetic patients. The resulting metabolites disrupt the functions of macrophages, endothelial cells and fibroblasts by promoting the secretion of inflammatory factors, inhibiting the secretion of VEGF and activating oxidative stress signaling. Collectively, these effects cause persistent inflammation, impaired angiogenesis and slow nerve regeneration. Therefore, diabetic wounds are characterized by a significantly prolonged inflammatory phase, which inhibits the normal wound healing process. Numerous studies have shown that wound healing can be significantly promoted by inhibiting the inflammatory state of the wound and promoting its progression to the repair phase [3–5].

Pyroptosis is a type of cell death mediated by the gasdermins, which include GSDMA/B/C/D/E (gasdermin A/B/C/D/E) and DFNB59 (Pejvakin, PJVK). Except for DFNB59, the gasdermins include two types of conserved structural domains, namely the N-terminal pore-forming structural domain and the C-terminal inhibitory structural domain. The N-terminus can form pores in cell membranes through oligomerization, triggering cellular pyroptosis and the release of large amounts of inflammatory factors [6]. In diabetic individuals, advanced glycation end products (AGEs) activate the pyroptosis pathway via the ROS/NLRP axis, resulting in delayed corneal wound healing and impaired nerve regeneration [7]. Recurrent Staphylococcus aureus infections activate the pyroptosis pathway, resulting in increased wound inflammation and delayed wound healing [8]. In addition, inhibition of the classical pyroptosis pathway significantly reduces the formation of neutrophil extracellular traps in diabetic wounds, thereby promoting healing [9]. The severe inflammation caused by pyroptosis contributes to the delayed healing of diabetic wounds [10].

Macrophages play an important role in wound healing. In the inflammatory phase, they primarily exist as M1-type macrophages, which recruit inflammation-related cells, release pro-inflammatory factors (TNF-α, IL-1β, IL-6, etc.), recognize and remove foreign pathogens and necrotic tissues and induce the polarization of other macrophages to the M1-type through signaling pathways such as NF-κB. During the proliferation and remodeling phase, macrophages primarily exhibit the M2 phenotype, which downregulates the secretion of inflammatory factors, promotes the expression of anti-inflammatory factors and vascular endothelial growth factor, and promotes angiogenesis and fibroblast proliferation and migration. Targeting macrophages can significantly improve the wound healing process by inhibiting diabetic wound inflammation and macrophage pyroptosis [1, 11–14]. Linagliptin is a dipeptidyl peptidase-4 (DPP4/CD26) inhibitor that can modulate the behavior of a variety of cells, including T-cells, macrophages, fibroblasts and keratinocytes. Therefore, a growing number of studies have focused on exploring the role and mechanisms of such drugs in wound healing, including diabetic ulcers and hypertrophic scar formation [15, 16].

Current research is focused on exploring more efficient medical dressings to promote wound healing by providing effective substances to localized wounds [17]. Nanofiber scaffolds prepared by electrostatic spinning technology have low side effects, stable drug releasing and high drug loading capacity. Moreover, their high variability in three-dimensional structure can also play a role as an extracellular matrix in wound healing. Many studies have confirmed that wound matrix-inducing membranes prepared using different polymers or natural macromolecules can be used as excellent carriers for drug loading and play a significant role in wound healing [18–20]. Therefore, the present study employed electrostatic spinning technology to prepare a wound matrix-inducing membrane with control released linagliptin. Based on a review of the relevant literature [13, 17–19], we selected PLGA [poly(lactic-co-glycolic acid)] and SF (silk fibroin) as the drug carrier matrix for this study. PLGA offers adjustable biodegradability and strong mechanical support, which serves as physical barrier for wounds. However, its hydrophobicity can inhibit early cell attachment and induce inflammation in tissue. To improve the biocompatibility, we incorporated SF, a natural protein with low immunogenicity and extracellular matrix-like structure. SF improves surface hydrophilicity and provides a favorable interface for cell proliferation. Moreover, combining SF with PLGA allows suitable sustained drug release. This mixture mitigates the initial burst release which is typical in hydrophilic systems and avoiding excessively slow diffusion in purely hydrophobic systems. The membrane was applied to a chronic wound animal model and effectively promoted wound healing.

## Materials and methods

### Materials

We provide the information of key materials in Supplementary Table S1.

### Cell culture and animals

L929, HUVEC, HaCaT and Raw 264.7 cells were cultured in high sugar DMEM medium. The medium was supplemented with 10% fetal bovine serum (FBS) and 1% penicillin-streptomycin. All cells were cultured at 37°C and 5% CO_2_.

Diabetic rats (purchased from Shanghai Cowen’s Biotechnology Co., Ltd.) were used to construct a diabetic wound model in this study. The *in vivo* study was performed following the guidelines established by the National Institutes of Health (NIH) for the care and use of laboratory animals (NIH Publication No. 85-23 Rev. 1985). The study protocol was approved by the Research Center for Laboratory Animals of the Navy Medical University of China [NMUMREC-2021-002].

### Preparation of PLGA/SF and PLGA/SF/linagliptin wound matrix-inducing membranes

To prepare the PLGA/SF films, 1 g PLGA and 0.3 g SF were dissolved in HFIP (Hexafluoroisopropanol) and stirred for 24 h as the material spinning solution. To prepare PLGA/SF/Linagliptin films with different drug mass fractions, linagliptin was mixed with HFIP and then sonicated. Subsequently, PLGA and SF were added according to the following ratios. PLGA/SF/Linagliptin 1: PLGA 1 g, SF 0.3 g and linagliptin 6.5 mg; PLGA/SF/Linagliptin 2: PLGA 1 g, SF 0.3 g and linagliptin 13 mg. Thereafter, the prepared spinning solution was transferred to a 10 mL syringe for electrostatic spinning. The temperature was set at 25–30°C, with a relative humidity of 60% and a flow rate of 1 mL/h; the voltage between the needle and the collector was set at 11–12 kV, and the distance was 15 cm and the drum collector rotated at 350 rpm. After electrostatic spinning, all the nanofibrous membranes were peeled off from the foils and dried in a vacuum to remove the residual HFIP.

### Characterization of PLGA/SF and PLGA/SF/linagliptin wound matrix-induced membranes

The morphology of both nanofiber membranes (samples pre-coated with gold) was observed by scanning electron microscopy under high vacuum conditions (accelerating voltage of 5–20 kV).

Differential scanning calorimetry (DSC) tests were carried out on a DSC2500 spectrometer (TA, USA). The DSC tests were performed in a nitrogen atmosphere at a gas flow rate of 250 mL/min. The sample powder (2–3 mg) was placed in a sealed aluminum crucible and heated in the range of 50–250°C at a rate of 10°C/min.

The mechanical properties of the nanofiber membranes were tested by a universal material testing machine (Autograph AGS-X, Japan) at room temperature. The electrospun membranes were cut into rectangular samples of 50 mm × 10 mm and stretched at a speed of 5 mm/min. Young’s modulus, breaking strength and elongation at break were calculated as the average of at least three test samples.

The hydrophilicity of the nanofiber membrane was assessed by the water contact angle (WCA). The samples were cut into small strips (30 mm × 10 mm) and pasted on slides. A camera was used to record the droplet’s interaction with the membrane, and a picture was taken every second.

Fourier-transform infrared spectroscopy (FTIR) was employed to determine the material composition and analyze conformational changes of the membranes, with scanning performed over a wavenumber range of 4000–600 cm^−1^. The crystal structure of the prepared samples was examined using X-ray diffraction (XRD), with a scanning Bragg angle (2θ) range of 5–90°. A copper target was used as the X-ray source, operating at 38 kV and 28 mA, and the scan rate was set at 2°/min to ensure sufficient signal resolution.

### Drug release assessment

In the drug release profile determination experiments of wound matrix-induced membranes, the films (400 ± 5 mg) were immersed in 4 mL of PBS buffer (pH = 7.4) and shaken at 37°C at 150 rpm. At specific time points, 2 mL of release solution was removed for absorbance measurement at a wavelength of 300 nm, and an equal amount of fresh PBS was added to keep the volume of the solution constant. At least three replicate experiments were set up for each group. The cumulative drug release percentage (CDR) was calculated according to the following formula:


CDR (%) = [mt/M] × 100%,

where mt and M denote the mass of drug released and the total mass of drug released at the time point of measurement, respectively. The calculation formula of drug encapsulation efficiency is


DEE (%) = Actual weight of Linagliptin in fiber/Theoretical weight of Linagliptin*100%.

### Biocompatibility testing

PLGA/SF and PLGA/SF/Linagliptin wound matrix-inducing membranes were immersed in 4 mL of DMEM medium for 72 h to prepare the material extracts.

In hemolysis test, venous blood was collected from mice and processed to prepare erythrocyte suspensions. The hemolysis assay included three groups: a negative control (PBS solution), a positive control (0.1% Triton X-100), and the experimental group treated with material extracts (EXT_1: the material extracts of PLGA/SF, EXT_2: the material extracts of PLGA/SF/Linagliptin). Each sample was thoroughly mixed with the erythrocyte suspension and incubated at 37°C with gentle shaking for 1 h after incubation, the mixtures were centrifuged at 1500 *g* for 10 min. The absorbance of the supernatant was then measured at 541 nm to assess hemolytic activity. Then, mouse peritoneal macrophages (Raw 264.7) were inoculated in 24-well plates at a density of 6 × 10^4^ cells/well, and cell activity was detected using the CCK-8 kit (Biosharp, China) after incubating the cells with two of the above-prepared extracts for 24 h and 48 h, respectively. L929 and HUVEC cells were digested from the dishes, and the cells were resuspended in the material extracts. The two types of cells were inoculated in 24-well plates at a density of 3 × 10^4^ cells/well, and incubated for 24 h. Subsequently, cytoskeletal staining was carried out using ghost pen cyclic peptide, and the morphology of the cells was observed.

### Cell viability assay

Human vascular endothelial cells (HUVEC), human immortalized keratinocytes (Hacat), mouse fibroblasts (L929) and mouse peritoneal macrophages (Raw 264.7) were inoculated in 24-well plates at a density of 3 × 10^4^ cells/well. The cells were allowed to adhere to the wall, and group interventions were conducted using linagliptin. The cell activity of each group was detected using the CCK-8 kit (Biosharp, China) after 24 and 48 h. Mouse peritoneal macrophages (Raw 264.7) were inoculated in 24-well plates at a density of 6 × 10^4^ cells/well, and cobalt chloride and glucose were added to the medium to simulate a high-glucose hypoxic environment, yielding a cobalt chloride concentration of 200 μM and a glucose concentration of 33 mM. After the cells had adhered to the wall, linagliptin was administered at different concentrations based on the groups. After 48 h, the cell activity of each group was detected using the CCK-8 kit (Biosharp, China).

### Cell migration assay

Mouse bone marrow-derived macrophages (BMDM) were inoculated at a density of 1 × 10^5^ cells per well in a 24-well plate. After 24 h of group intervention, the cell culture supernatant was collected and reserved for later use. HUVEC and L929 cells were then inoculated into the upper chamber of the Transwell chamber. The cells were allowed to adhere to the chamber, and the previously collected cell supernatant was added to the 24-well plate below the Transwell chamber. After 24 h of group intervention, the upper chamber of the Transwell was removed. The cells were fixed and stained with crystal violet and counted under a microscope.

### Immunoblotting experiment

Protein sample preparation: treated cell or tissue samples were collected, and RIPA lysis buffer (containing 1% PMSF) was added. The samples were lysed on ice for 30 min, centrifuged at 14 000 rpm for 20 min at 4°C, and the supernatant was collected. To determine the protein concentration by the BCA(bicinchoninic acid) method, all samples were adjusted to a uniform concentration with the uploading buffer, and the proteins were denatured by boiling at 70°C for 10 min. For gel electrophoresis, aliquots of protein samples were loaded onto a 4–12% Bis-Tris precast gel (GenScript, China) and electrophoresed at a constant voltage of 170 V. Electrophoresis was terminated when the bromophenol blue indicator reached the bottom of the gel. Membrane transfer: The wet transfer method was used to transfer the protein to a PVDF (polyvinylidene fluoride) membrane. Membrane transfer was performed under a constant current of 260 mA for 120 min at 4°C. After membrane transfer, the membrane was closed with 5% skimmed milk for 2 h at room temperature. Antibody incubation: The closed PVDF membrane was washed with TBST solution, followed by overnight incubation at 4°C with the target primary antibodies. On the next day, the samples were incubated with the corresponding HRP-labeled secondary antibodies (1:10 000 dilution ratio) for 2 h at room temperature. Development and quantification: the ECL chemiluminescence kit was used for development, and the signal was acquired from the ChemiDoc imaging system.

### Immunofluorescence

Sample preparation: Raw 264.7 cells were inoculated in 24-well plates at a density of 6 × 10^4^ cells/well, wall-plated, and then, grouped for intervention. After 48 h, the cells were fixed using 4% paraformaldehyde for 30 min at room temperature and washed three times with PBS. Thereafter, 0.3% Triton was added for 15 min for fixation and washed 3 times with PBS. Blocking was performed with 5% BSA for 2 h at room temperature to reduce nonspecific binding. Subsequently, diluted primary antibodies (dilution: 3% BSA) were added dropwise and incubated at 4°C overnight. The next day, fluorescent-labeled secondary antibodies (1:1000) were added dropwise under light-avoidance conditions and incubated for 2 h at room temperature away from light. Nuclear staining: DAPI was added dropwise and incubated for 1 minute at room temperature away from light. Images were acquired using a fluorescence microscope with fixed exposure parameters.

### Electron microscopy scanning

Mouse bone marrow-derived macrophages (BMDM) were inoculated at a density of 8 × 10^5^ cells/well in a 6-well plate with pre-positioned coverslips, and after apposition, they were grouped for intervention. After 24 h, they were rinsed 3 times using PBS, and then, fixed with electron microscopy fixative for 2 h at room temperature and sent to Ningbo Yangming Medical Laboratory Inc. for scanning electron microscopy of cell morphology.

### Cell supernatant Elisa assay

Mouse bone marrow-derived macrophages (BMDM) were inoculated in 6-well plates at a density of 1 × 10^6^ cells/well and allowed to adhere to the walls, and grouped interventions were carried out accordingly. After 24 h, the cell supernatant was collected, and the IL-1β ELISA kit (ABclonal, China) was utilized following the manufacturer’s instructions.

### Diabetic wound healing experiment *in vivo*

Diabetic rats (purchased from Shanghai Cowen’s Biotechnology Co., Ltd.) were used in this study and were randomly divided into 3 groups (*n* ≥ 3 in each group), including the control group (untreated), the PLGA/SF group and the PLGA/SF/Linagliptin group.

Modeling: rats were anesthetized using isoflurane gas, and bilateral full-thickness circular wounds of 15 mm diameter were created on their backs. Different dressings were given according to the grouping.

Healing assessment and sample collection: Wounds were photographed and recorded at 0, 7, 14 and 17 days after surgery, and the area of the wounds was calculated using ImageJ software. Healing rate calculation formula: Healing rate (%) = (initial wound area—wound area on the evaluation day)/initial wound area × 100%.

Tissue analysis: rats were executed on Days 7, 14 and 17, and tissues from the operated area were collected for subsequent histological testing (H&E fluorescent staining, etc.) and RNA sequencing (RNA-seq).

### Histological testing

The healing tissue samples were collected from the rats’ backs and were fixed with 4% paraformaldehyde (Beyotime, China), then subjected to gradient dehydration, followed by paraffin-embedding. Subsequently, a paraffin slicer (Leica RM2265) was used to section the samples.

Staining protocol: H&E staining was performed to assess the morphological characteristics of tissues. Masson trichrome staining was conducted to detect the degree of collagen deposition and fibrosis. Immunofluorescence staining: CD163, CD31, α-SMA, Col I, Col III, MPO.

### RNA-seq of animal tissue samples

The rats were executed on the seventh day of modeling, and the dorsal wound tissues were taken, which were divided into three groups: 1. control group (Con); 2. PLGA/SF group (Mat); and 3. PLGA/SF/Linagliptin group (Lin), with three samples in each group. Subsequently, the samples were snap-frozen in liquid nitrogen and then sent to Suzhou GENEWIZ Biotechnology Co. for RNA-seq analysis. Differentially expressed genes (DEGs) were analyzed and annotated with the KEGG database and the GO database.

### Statistical methods

The results were labeled using the mean ± SEM of repeated experiments. For significance testing, the t-test was used for two-group comparisons; one-way ANOVA analysis was used for multiple-group comparisons (significance threshold: *P* < 0.05).

## Results

### The effect of linagliptin on wound healing-related cells

#### Effect of linagliptin intervention on cellular activity

**Figure 1:**
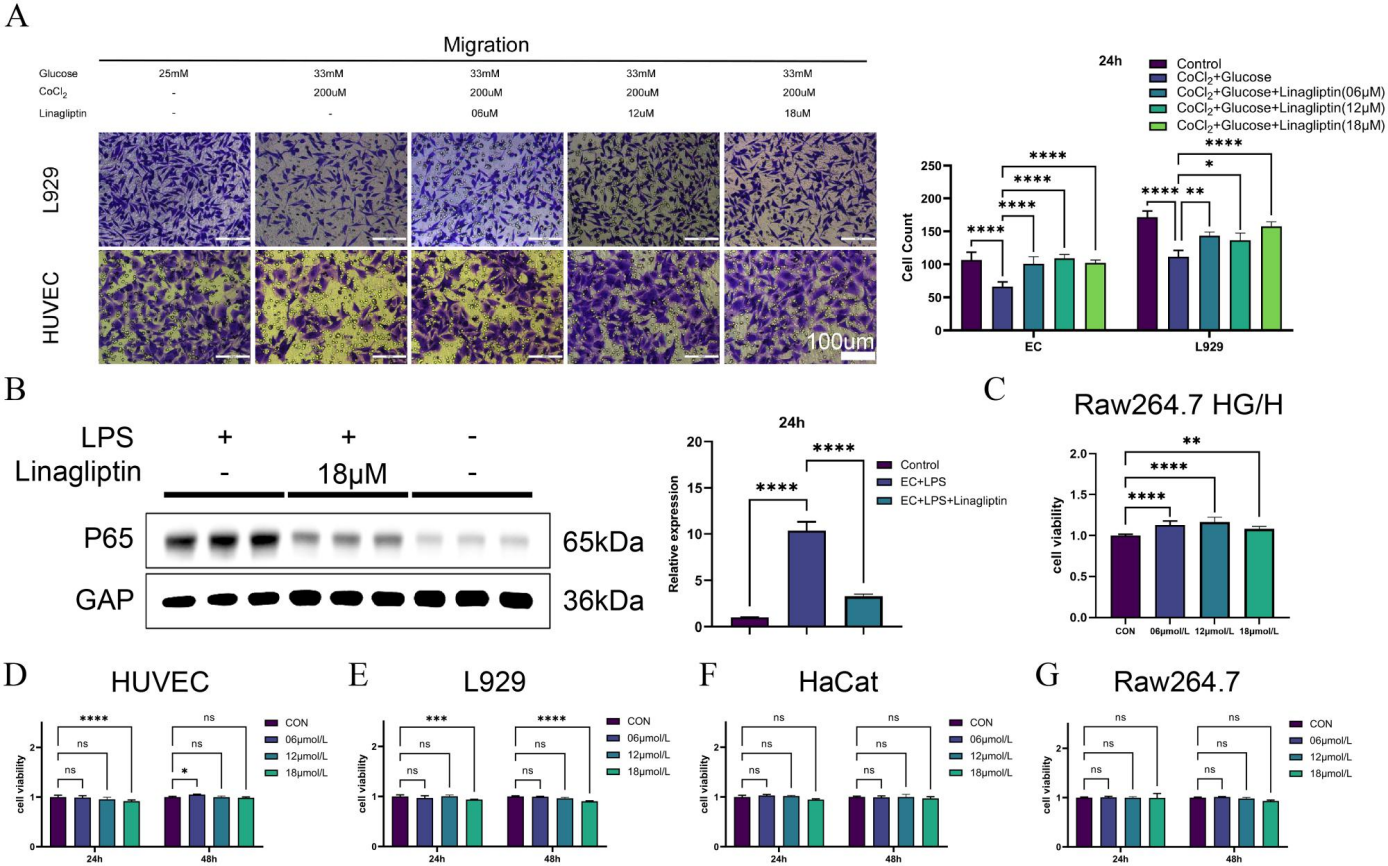
Effects of linagliptin intervention on cells related to wound healing. (**A**) The transwell assay evaluated the migration capacity of HUVECs and L929 cells using the supernatant obtained from treating BMDMs with linagliptin under high-glucose/hypoxic (HG/H) conditions. (**B**) Western blot analysis detected the expression of inflammation-related proteins in endothelial cells under different experimental conditions. (**C**) Under high-glucose/hypoxic (HG/H) conditions, linagliptin was administered to Raw 264.7 cells and cell viability was measured using the CCK8 assay. (**D**–**G**) Linagliptin was administered to various cells related to wound healing, and cell viability was measured using the CCK8 assay. ns: *P* > 0.05, *: *P* ≤ 0.05, **: *P* ≤ 0.01, ***: *P* ≤ 0.001, ****: *P* ≤ 0.0001.

The cells were treated with different concentrations of linagliptin solution for 24 h and 48 h, and the CCK-8 colorimetric assay was employed to detect the cell activity, as shown in figures (Figure 1C–G). For HaCaT and Raw 264.7, the cell activity of each group showed no significant differences. For HUVEC, at the 24 h time point and a drug concentration of 18 μM, a slight reduction in cell viability was observed; however, viability remained above 90%. Extending the intervention to 48 h, no significant decrease in cell viability across all experimental groups was observed. For L929, at the both time points and a drug concentration of 18 μM, a slight reduction in cell viability was observed; however, viability remained above 90%. Under the high-glucose/hypoxic (HG/H) conditions, cell viability was increased after different concentrations of linagliptin solution were added to treat Raw 264.7 cells for 48 h.

#### Linagliptin affects other cell migration via macrophages in high-glucose/hypoxic (HG/H) conditions

In this experiment, different concentrations of linagliptin were added to the culture medium, simulating the diabetic wound microenvironment. The migration function of L929 and HUVEC was inhibited, and the number of migrated cells was significantly lower than that of the control group under the high-glucose/hypoxic (HG/H) conditions. In contrast, the migration function of L929 and HUVEC was enhanced following linagliptin addition to treat BMDM. This effect did not change significantly with the increase in the concentration of the linagliptin solution. This demonstrated that BMDM treated with linagliptin significantly improved the migration function of L929 and HUVEC through paracrine effects under high-glucose/hypoxic (HG/H) conditions (Figure 1A).

#### Inhibition of cellular inflammation-related phenotypes by linagliptin

In this experiment, an endothelial cell inflammation model was constructed using LPS, and the cells were treated with different concentrations of linagliptin. The high expression of P65 in endothelial cells was induced by LPS, whereas the expression of this protein was significantly inhibited by the addition of linagliptin treatment (Figure 1B).

### Construction and characterization of PLGA/SF/linagliptin wound matrix-induced membrane

**Figure 2:**
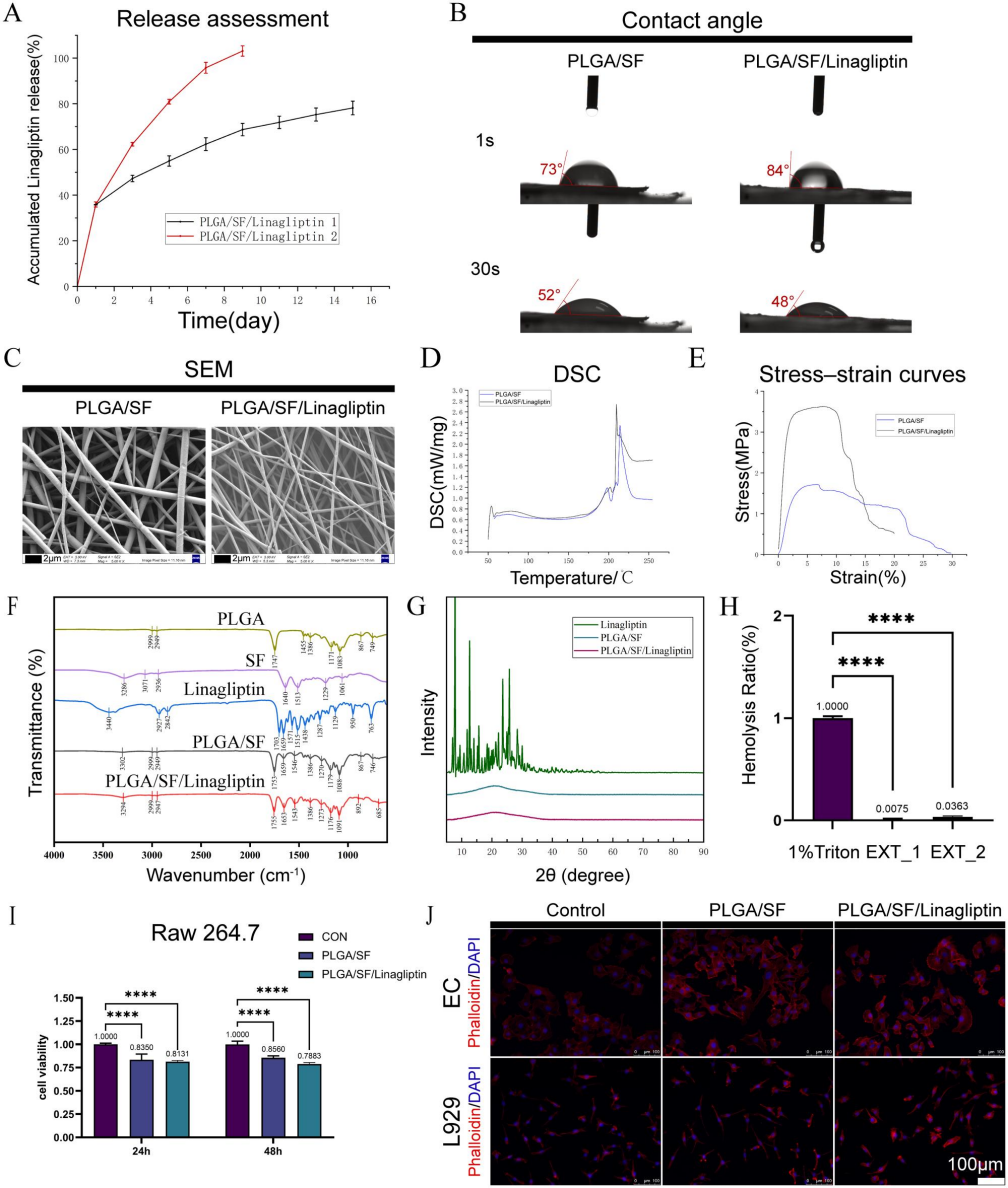
Material characterization of PLGA/SF wound matrix-induced membranes and PLGA/SF/linagliptin wound matrix-induced membranes. (**A**) Drug release curves of PLGA/SF/linagliptin 1 and PLGA/SF/linagliptin 2 wound matrix-induced membranes, measured over 0–15 days. (**B**) Hydrophilic contact angles of PLGA/SF and PLGA/SF/linagliptin wound matrix-induced membranes upon contact with PBS solution for 1 s and 30 s. (**C**) Scanning electron microscopy (SEM) morphology of PLGA/SF and PLGA/SF/linagliptin wound matrix-induced membranes. (**D**) DSC curves of PLGA/SF and PLGA/SF/linagliptin wound matrix-inducing membranes. (**E**) Stress–strain curves of PLGA/SF and PLGA/SF/linagliptin wound matrix-inducing membranes. (**F**) The FTIR spectra of all components and membranes. (**G**) The XRD of linagliptin, PLGA/SF and PLGA/SF/linagliptin wound matrix-inducing membranes. (**H**) The hemolysis test of the material extracts (EXT_1: the material extracts of PLGA/SF, EXT_2: the material extracts of PLGA/SF/linagliptin). (**I**) Cell viability of Raw 264.7 cells under different extract solution from the PLGA/SF and the PLGA/SF/linagliptin wound matrix-induced membrane. (**J**) Cell morphology of L929 and EC cells after 24 h of culture with the extract solutions from the PLGA/SF and the PLGA/SF/linagliptin wound matrix-induced membranes. ns: *P* > 0.05, *: *P* ≤ 0.05, **: *P* ≤ 0.01, ***: *P* ≤ 0.001, ****: *P* ≤ 0.0001.

In this study, two PLGA/SF/linagliptin wound matrix-inducing membranes with different drug mass fractions (PLGA/SF/linagliptin 1 and PLGA/SF/linagliptin 2) were prepared, and their drug delivery profiles were determined. As shown in Figure 2A, the PLGA/SF/Linagliptin 1 wound matrix-induced membrane showed a more stable rate of drug release, which continuously and gradually released the drug for more than 15 days. Specifically, the drug was released into PBS solution at a faster rate in the first 3 days of the experiment, and then, a more stable release rate was maintained in the following 2 weeks. In contrast, the PLGA/SF/Linagliptin 2 wound matrix-inducing membrane released about 99% of the loaded drug within 1 week, and did not achieve a long-term stable drug release. Therefore, PLGA/SF/Linagliptin 1 wound matrix-inducing membrane was used in the subsequent material science and biology experiments. And based on this result, we calculate the DEE (drug encapsulation efficiency) to be approximately 82%. Then, the hydrophilicity of both PLGA/SF and PLGA/SF/Linagliptin films was measured, as shown in Figure 2B. At the 1 s time point, the hydrophilic contact angle of PLGA/SF membrane is 73°, the hydrophilic contact angle of PLGA/SF/Linagliptin membrane is 84°. At the 30 s time point, the hydrophilic contact angle of PLGA/SF membrane is 52°, the hydrophilic contact angle of PLGA/SF/Linagliptin membrane is 48°.

The morphology of PLGA/SF and PLGA/SF/Linagliptin electrostatic spinning membranes was observed by scanning electron microscopy (SEM), as displayed in Figure 2C. Both membranes demonstrated a loose and porous internal structure, showing some variation in fiber diameter, which was attributed to the uneven dissolution of SF in isopropylhexafluoride and the insufficient viscosity of the solution. Compared with PLGA/SF electrostatic spinning membranes, linagliptin addition caused changes in some of the fiber diameters, which were attributed to its strong hydrophobicity. The DSC thermograms (Figure 2D) revealed that both electrostatic spinning membranes exhibited a heat-absorbing melting peak, indicating that linagliptin may be amorphous in the electrostatically spun fibers. Among them, the PLGA/SF film showed a heat absorption melting peak onset of 211.5°C, an end point of 220.7°C and an area of 121.9 J/g. In addition, the PLGA/SF/Linagliptin showed a heat absorption melting peak onset of 209.7°C, an end point of 211.1°C and an area of 83.9 J/g. The incorporation of linagliptin did not significantly affect the thermal properties of PLGA/SF copolymer. Finally, the mechanical properties of the prepared fiber membranes were evaluated, as shown in Figure 2E. The maximum tensile stresses that PLGA/SF wound matrix-induced membranes could withstand ranged from 1.7 to 2.7 MPa; the Young’s modulus ranged from 77 to 114 MPa, and the elongation at break ranged from 6.5% to 18%. The maximum tensile stresses that the PLGA/SF/Linagliptin wound matrix-induced membranes could withstand ranged from 3.2 to 3.6 MPa, with a Young’s modulus range of 204–244 MPa and an elongation at break range of 5–8%.

As for the FTIR and XRD results (Figure 2F and G), pure PLGA displayed characteristic absorption peaks at 2999 cm^−1^, 2949 cm^−1^, 1747 cm^−1^, 1455 cm^−1^, 1386 cm^−1^, 1171 cm^−1^, 1083 cm^−1^, 867 cm^−1^ and 749 cm^−1^, all peaks are consistent with the linear polymeric structure of PLGA. Similarly, pure silk fibroin (SF) showed peaks at 3286 cm^−1^, 3071 cm^−1^, 2936 cm^−1^, 1640 cm^−1^, 1513 cm^−1^, 1229 cm^−1^ and 1061 cm^−1^, which align closely with reference spectra reported for SF. In the PLGA/SF membranes, each observed peak could be clearly attributed to either PLGA or SF, with no significant shift or disappearance of the characteristic PLGA absorptions. This indicates that no pronounced chemical interaction occurred between the two components during fabrication. Notably, in the PLGA/SF/Linagliptin membranes, we did not detect distinct FTIR peaks corresponding to linagliptin. This is likely due to its relatively low weight in the membranes and the overlap of its characteristic bands with the strong absorption signals from PLGA. The results of XRD experiments are shown above, pure linagliptin exhibited characteristic diffraction peaks at multiple positions, including 2θ  =  7.82°, 12.64°, 25.76°, 23.58°, 28.4° and 21.28°. In contrast, no such distinct crystalline peaks were observed in the samples of the PLGA/SF and PLGA/SF/Linagliptin membranes. This absence indicates that the drug is homogeneously dispersed within the composite system in an amorphous state, which we attribute to its molecular-level integration during the electrospinning process.

To further investigate the biocompatibility of the material, the extracts of the two materials were prepared. The hemolysis test exhibited that both material extracts does not cause significant hemolysis (Figure 2H). Raw 264.7 cells were cultured in these extracts, respectively, and the cell viability was determined by the CCK8 assay after 24 h and 48 h. Cell viability was somewhat inhibited by the extracts of both PLGA/SF film and PLGA/SF/Linagliptin film compared to the control, but the cell survival rate remained >75% in both groups (Figure 2I). According to ISO 10993-5, this suggests that the above two materials did not exhibit significant biotoxic effects. Finally, the extracts were used to culture endothelial cells and fibroblasts to explore their effects on cell adhesion. The results of cytoskeletal staining revealed no significant differences in cellular morphology across all experimental groups compared to the control (Figure 2J).

### Inhibition of macrophage pyroptosis and its phenotypic transformation by linagliptin in a hyperglycemic hypoxic environment

**Figure 3:**
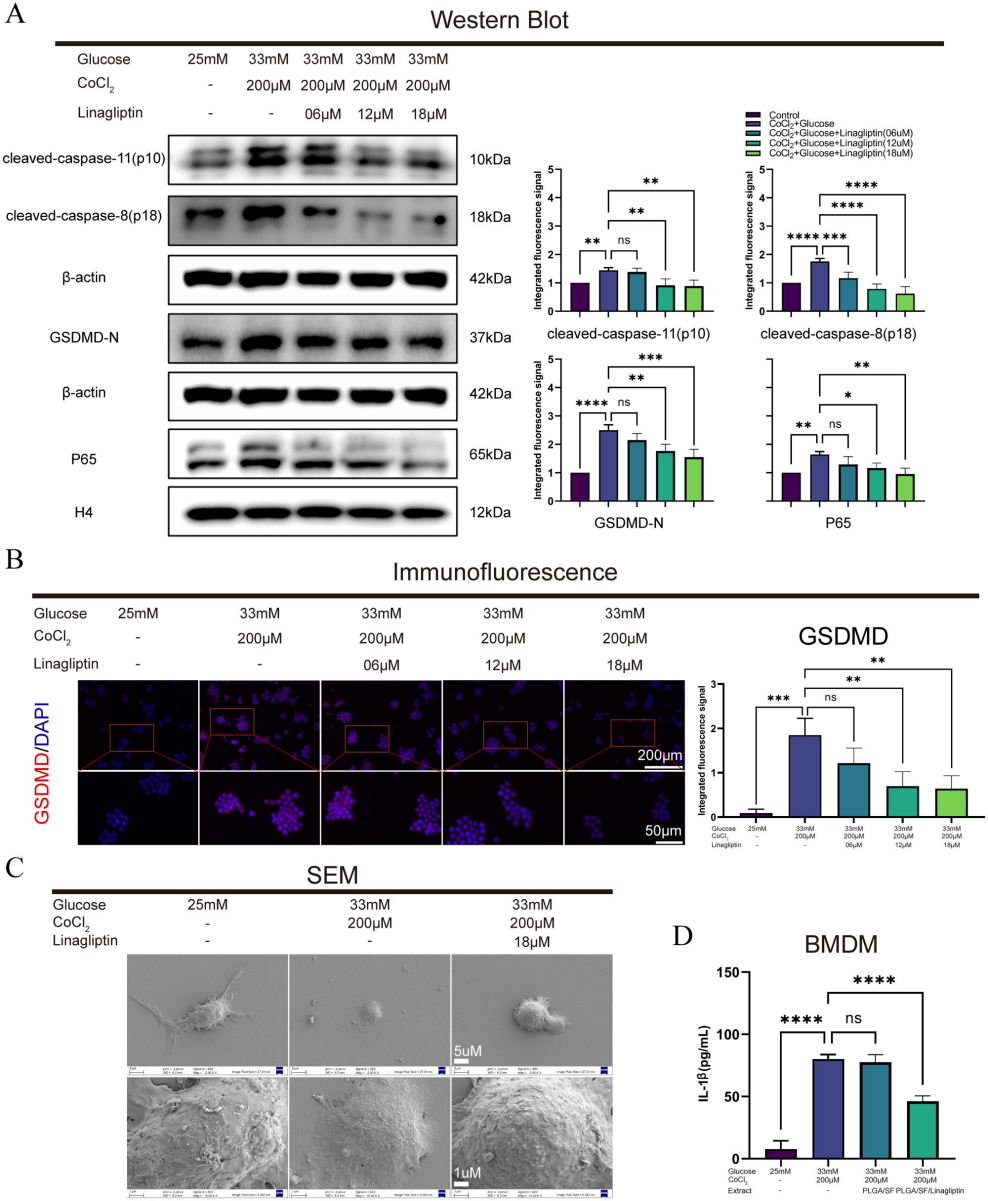
Under high-glucose/hypoxic (HG/H) conditions, linagliptin inhibits macrophage pyroptosis. (**A**) After treating Raw 264.7 cells with different concentrations of linagliptin for 48 h, the expression of pyroptosis pathway-related proteins was measured using Western blot analysis. (**B**) After treating Raw 264.7 cells with different concentrations of linagliptin for 48 h, the expression of pyroptosis pathway proteins was measured using immunofluorescence experiments. (**C**) After treating BMDMs cells with linagliptin (18 μM) for 24 h, cell morphology was observed using scanning electron microscopy. (**D**) After treating BMDM cells with two different material extracts for 24 h, the concentration of IL-1β in the supernatant was measured using an ELISA assay. ns: *P* > 0.05, *: *P* ≤ 0.05, **: *P* ≤ 0.01, ***: *P* ≤ 0.001, ****: *P* ≤ 0.0001.

To investigate the effect of linagliptin on macrophage pyroptosis and inflammation in diabetic wounds, molecular experiments revealed that nuclear p65 levels in Raw 264.7 cells were significantly elevated compared to the control group under high-glucose/hypoxic conditions. Treatment with various concentrations of linagliptin markedly reduced nuclear p65 accumulation, restoring its expression to levels compared with the control group (Figure 3A). The results of our immunoblotting experiments also demonstrated no significant change in the expression of NLRP3 in Raw 264.7 cells in the experimental group (Supplementary Figure S1).

Furthermore, the expression of its GSDMD N-terminal was significantly increased in a high-glucose hypoxic environment, and decreased after treatment with linagliptin. These findings suggest that GSDMD can be regulated through a non-caspase-1-dependent mechanism in Raw 264.7 cells (Figure 3A). Notably, the expression of cleaved-caspase-11 and cleaved-caspase-8 was significantly increased under high-glucose/hypoxic (HG/H) conditions, which decreased after linagliptin treatment (Figure 3A). These results demonstrated that linagliptin inhibited caspase-11 and cleaved-caspase-8 mediated GSDMD N-terminal, indicating involvement of a nonclassical pyroptosis pathway.

Immunofluorescence staining revealed a significant increase in GSDMD fluorescence intensity in Raw 264.7 cells under high-glucose/hypoxic (HG/H) conditions compared to controls. Treatment with linagliptin reduced this intensity in a concentration-dependent manner, as evidenced by progressively decreasing relative fluorescence intensity with higher linagliptin doses. This further demonstrates linagliptin’s ability to suppress HG/H-induced GSDMD overexpression in Raw 264.7 cells (Figure 3B).

Our SEM analysis revealed that bone marrow-derived macrophages (BMDMs) under normal conditions exhibited a plump morphology with intact membranes and multiple pseudopodia. In contrast, BMDMs exposed to HG/H conditions displayed collapse, lysis and irregular vesicles on the membrane surface, accompanied by loss of basic cellular structure. Linagliptin treatment ameliorated these morphological changes, preventing overt collapse and lysis and significantly reducing pyroptotic membrane features, although normal pseudopodia morphology was not fully restored (Figure 3C).

To further validate the anti-pyroptotic effect of the material extracts, IL-1β levels in cell supernatants were quantified by ELISA. BMDMs under HG/H conditions exhibited significantly increased IL-1β release. PLGA/SF extract treatment did not alter IL-1β concentrations, but PLGA/SF/Linagliptin extract significantly reduced IL-1β secretion (Figure 3D). These results confirm that the PLGA/SF/Linagliptin extract inhibits IL-1β release from BMDMs by drug elution.

### Role of PLGA/SF/linagliptin wound matrix-inducing membranes in diabetic rat wound models

#### PLGA/SF/linagliptin wound matrix-inducing membrane promotes wound healing in diabetic rats

**Figure 4:**
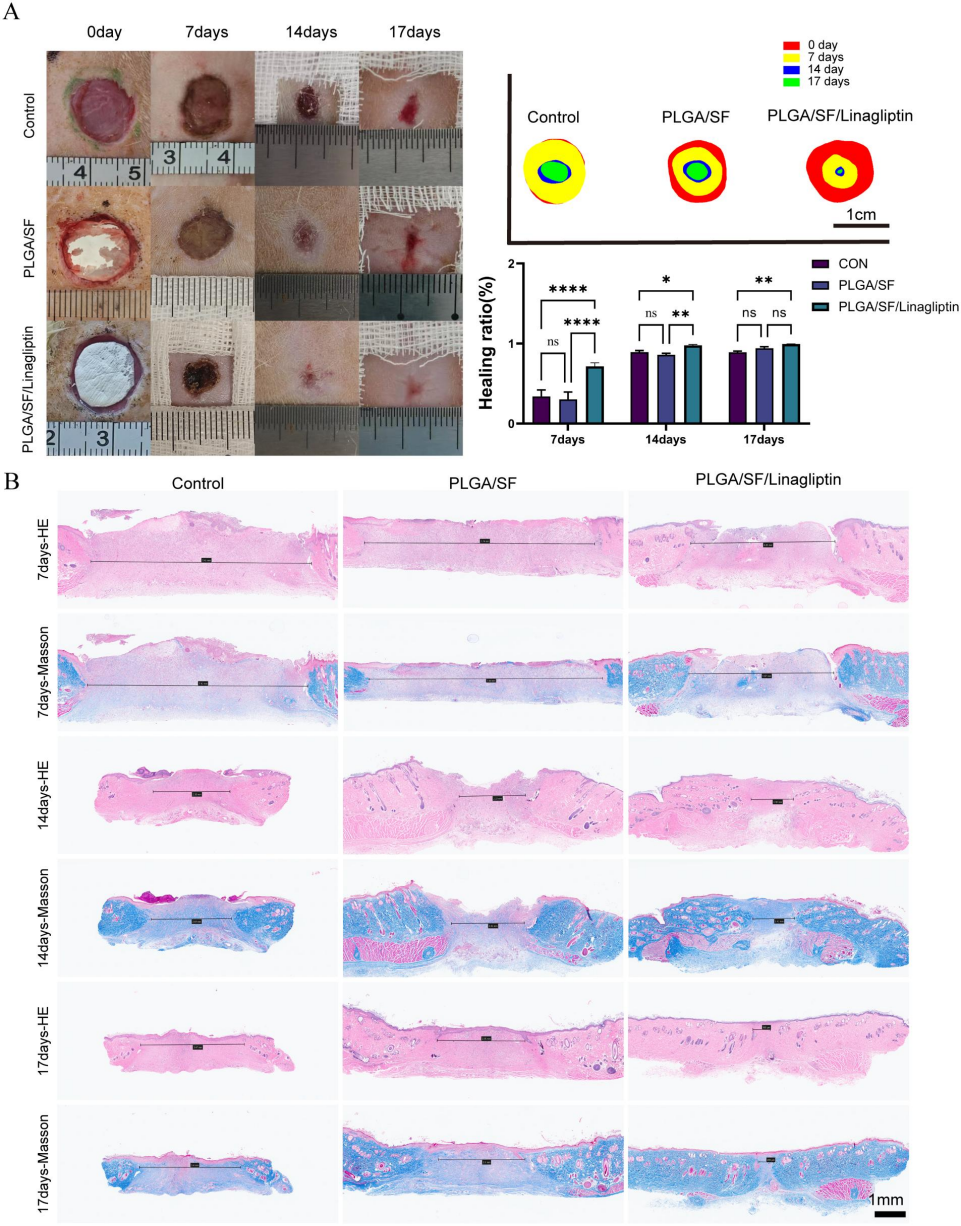
Effects of PLGA/SF/linagliptin and PLGA/SF wound matrix-induced membranes in a diabetic rat wound model. (**A**) Wound images and statistical graphs of experimental animals in different groups at 7, 14 and 17 days. (**B**) HE staining and Masson staining results of wound samples from experimental animals in different groups at 7, 14 and 17 days. ns: *P* > 0.05, *: *P* ≤ 0.05, **: *P* ≤ 0.01, ***: *P* ≤ 0.001, ****: *P* ≤ 0.0001.

*In vivo* experiments were conducted to further evaluate the effects of PLGA/SF/Linagliptin and PLGA/SF films on wound healing. Diabetic rats were utilized to construct a skin wound model, and wound healing was recorded on Days 0, 7, 14 and 17. The PLGA/SF/Linagliptin group exhibited the most significant effect on promoting wound healing. On Day 7, the wound epithelialization in this group had reached approximately 70%, which was significantly better than that of the PLGA/SF group (approximately 30%) and the control group (approximately 30%). On Day 14, the wound was already close to being fully epithelialized, while the PLGA/SF group and control group still had not completed the epithelialization process (Figure 4A).

Furthermore, by Day 7, the wound area in the PLGA/SF/Linagliptin group was significantly smaller compared to the other groups, which could be attributed to the linagliptin released from the material facilitating the transition of wound healing to the remodeling phase by inhibiting persistent inflammation. The above findings were verified by performing staining analysis on the tissue samples. Masson and H&E staining suggested that the epithelialization process was significantly accelerated in the PLGA/SF/Linagliptin group at all observed time points, with a significant increase in collagen fiber synthesis and subcutaneous tissue proliferation levels (Figure 4B).

**Figure 5:**
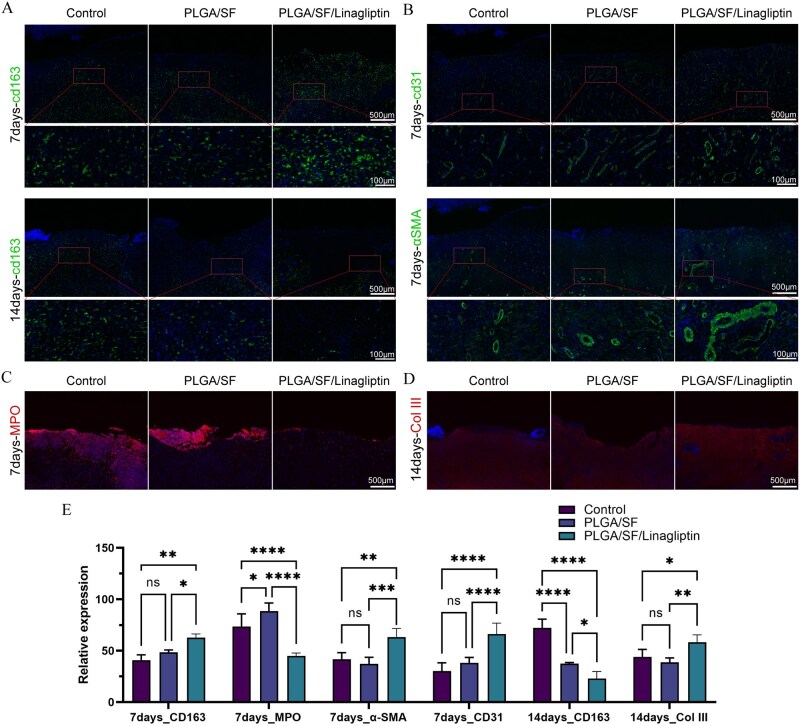
The effects of PLGA/SF/linagliptin and PLGA/SF wound matrix-induced membranes in a diabetic rat wound model. (**A**) CD163 immunofluorescence staining results at 7 and 14 days in different groups of experimental animals. (**B**) Immunofluorescence staining results for CD31 and α-SMA at 7 days in experimental animals from different groups. (**C**) Immunofluorescence staining results for MPO at 7 days in experimental animals from different groups. (**D**) Immunofluorescence staining results for Col III at 14 days in experimental animals from different groups. (**E**) Analysis of all the immunofluorescence staining results, in 7 days, the group of PLGA/SF/linagliptin exhibited lower level of MPO and higher level of CD163, CD31, α-SMA; in 14 days, the group of PLGA/SF/linagliptin exhibited lower level of CD163 and higher level of col III. ns: *P* > 0.05, *: *P* ≤ 0.05, **: *P* ≤ 0.01, ***: *P* ≤ 0.001, ****: *P* ≤ 0.0001.

Next, immunofluorescence staining was carried out on the tissue sections. On Day 7, a higher expression of CD163 and a lower expression of MPO were observed in the PLGA/SF/Linagliptin group compared with the other two groups. Additionally, the density of CD31+ neovascularization and the number of CD31+/α-SMA+ mature blood vessels in the PLGA/SF/Linagliptin group were significantly higher than those in the other two groups (Figure 5A–C).

#### Analysis of PLGA/SF/linagliptin wound matrix-induced membrane sequencing data

According to the results of the aforementioned molecular and histological experiments, the PLGA/SF/Linagliptin group exhibited lower expression of inflammation-related proteins compared with the other groups, and the sustained and stable release of linagliptin inhibited the activation of the pyroptosis pathway. To validate this differential expression at the RNA level, the variability of RNA expression in each group was analyzed using transcriptome sequencing (RNA-seq) technology to assess the effect of linagliptin on inflammation regulation and cellular pyroptosis.

**Figure 6:**
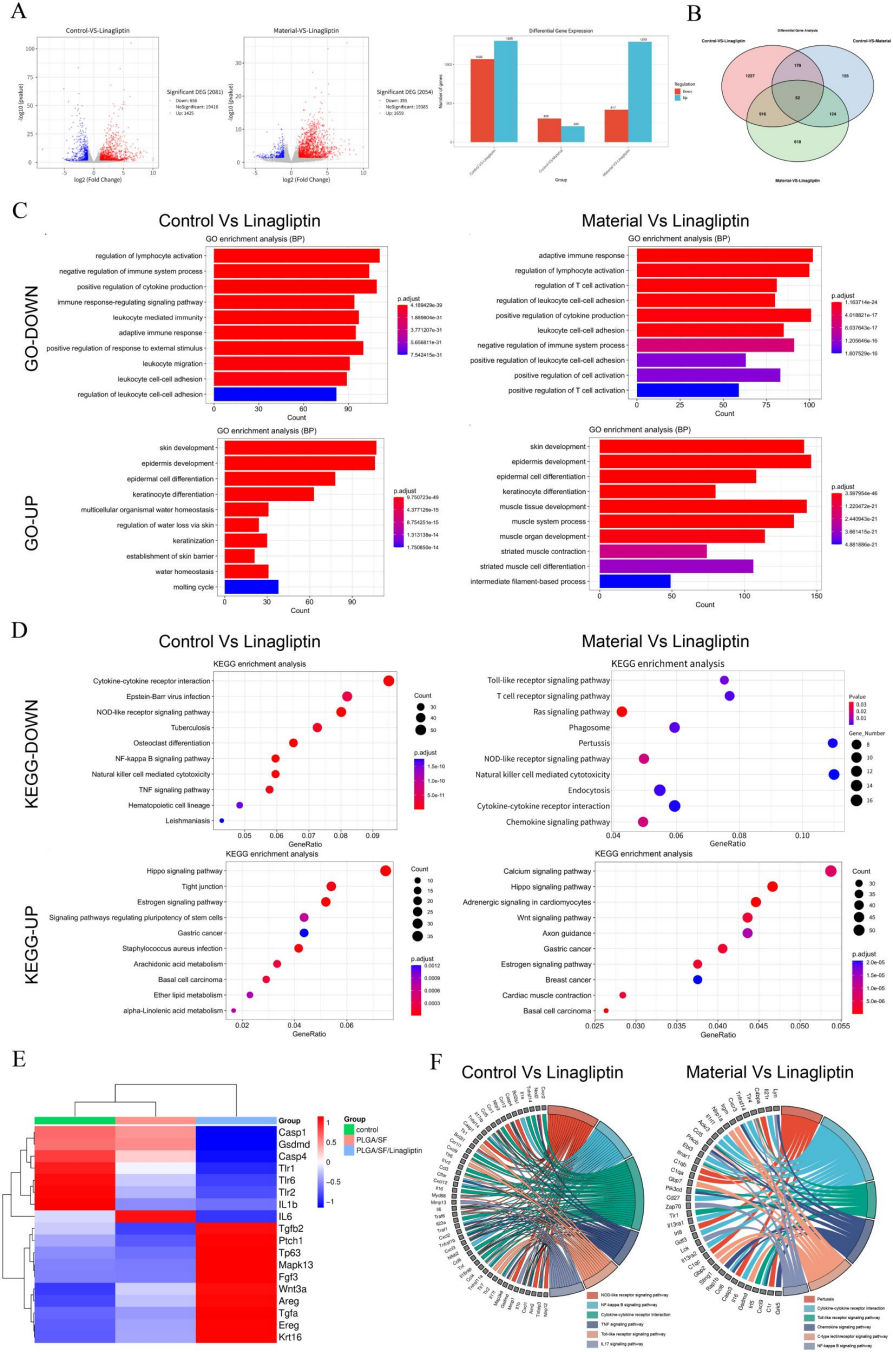
RNA-seq analysis of wound samples from different groups of animals (‘control’ represents the control group, ‘material’ represents the PLGA/SF group, ‘linagliptin’ represents the PLGA/SF/linagliptin group; ‘a vs B’ represents the gene expression of group B relative to group A, divided into upregulation and downregulation). (**A**) Volcano plot and bar chart of differentially expressed genes between different groups. (**B**) Venn diagram of differentially expressed genes when comparing different groups. (**C**) Visualization of differentially expressed genes enriched in GO (BP) when comparing different groups. (**D**) Visualization of differentially expressed genes enriched in KEGG when comparing different groups. (**E**) Visualization of differentially expressed genes related to wound healing (heatmap). (**F**) Visualization of downregulated genes enriched in specific pathways when comparing different groups (chord diagram).

To verify that linagliptin released from the PLGA/SF/Linagliptin film inhibited the pyroptosis pathway during wound healing, wound samples from the PLGA/SF/Linagliptin group, the PLGA/SF group and the control group on Day 7 of wound healing were subjected to transcriptome sequencing analysis. After quality control, the gene expression of the samples in each group was analyzed, paying particular attention to the expression of genes related to pyroptosis and inflammation in the different groups. Thereafter, the results were visualized by volcano plots, thermograms and GO and KEGG enrichment analyses (Figure 6C and D) to explore the differences in the expression of biological processes and gene pathways between the different groups.

The volcano map (|log2FC| > 0.585) demonstrated the distribution of DEGs that varied significantly among the three groups (Figure 6A). The heatmaps and chord diagrams show differential gene expression between the three groups (Figure 6E and F). The Venn diagram showed the number of genes with significant differential expression between groups (Figure 6B).

Compared with the control group, GO analysis revealed that upregulated DEGs in the PLGA/SF/Linagliptin group were enriched in skin epithelialization, keratinization, skin barrier generation, cell adhesion and junction. Moreover, downregulated DEGs were enriched in immune response, inflammatory response and cytokine production, etc. KEGG analysis showed that the Hippo signaling pathway, estrogen signaling pathway and tight junction-related signaling were significantly upregulated in the PLGA/SF/Linagliptin group compared with the control group. The significantly downregulated pathways included NOD-like receptor signaling pathway, NF-κB signaling pathway, TNF signaling pathway and cytokine-receptor interaction signaling, etc., which were significantly correlated with pyroptosis (Figure 6C and D). Compared with the PLGA/SF group, GO analysis revealed that the upregulated genes in the PLGA/SF/Linagliptin group were enriched in skin epithelialization, keratinization, skin barrier generation, cell junction formation, etc. The downregulated genes were enriched in leukocyte activation/proliferation/migration, inflammatory response and acute immune response. KEGG analysis indicated that the cytoskeleton-related signaling, Hippo signaling pathway, Wnt signaling pathway and calcium signaling pathway were significantly upregulated in the PLGA/SF/Linagliptin group compared with the PLGA/SF group. The significantly downregulated pathways included Toll-like receptor signaling pathway, NOD-like receptor signaling pathway, Ras signaling pathway and cytokine and receptor interaction signaling, etc., which were significantly correlated with pyroptosis (Figure 6C and D).

Clustering analysis of differential genes revealed that genes related to the pyroptosis pathway were significantly downregulated in the PLGA/SF/Linagliptin group compared to both the control group and the PLGA/SF group. In the PLGA/SF/Linagliptin group, the expression of genes related to the pyroptosis pathway and many inflammation-related genes was significantly suppressed by linagliptin, while the expression of several signaling pathways related to re-epithelialization and tissue regeneration was enhanced (Figure 6E). The differential expression of these genes suggests a molecular mechanism by which linagliptin promotes diabetic wound healing.

#### The expression differences of pyroptosis pathways *in vivo*

**Figure 7:**
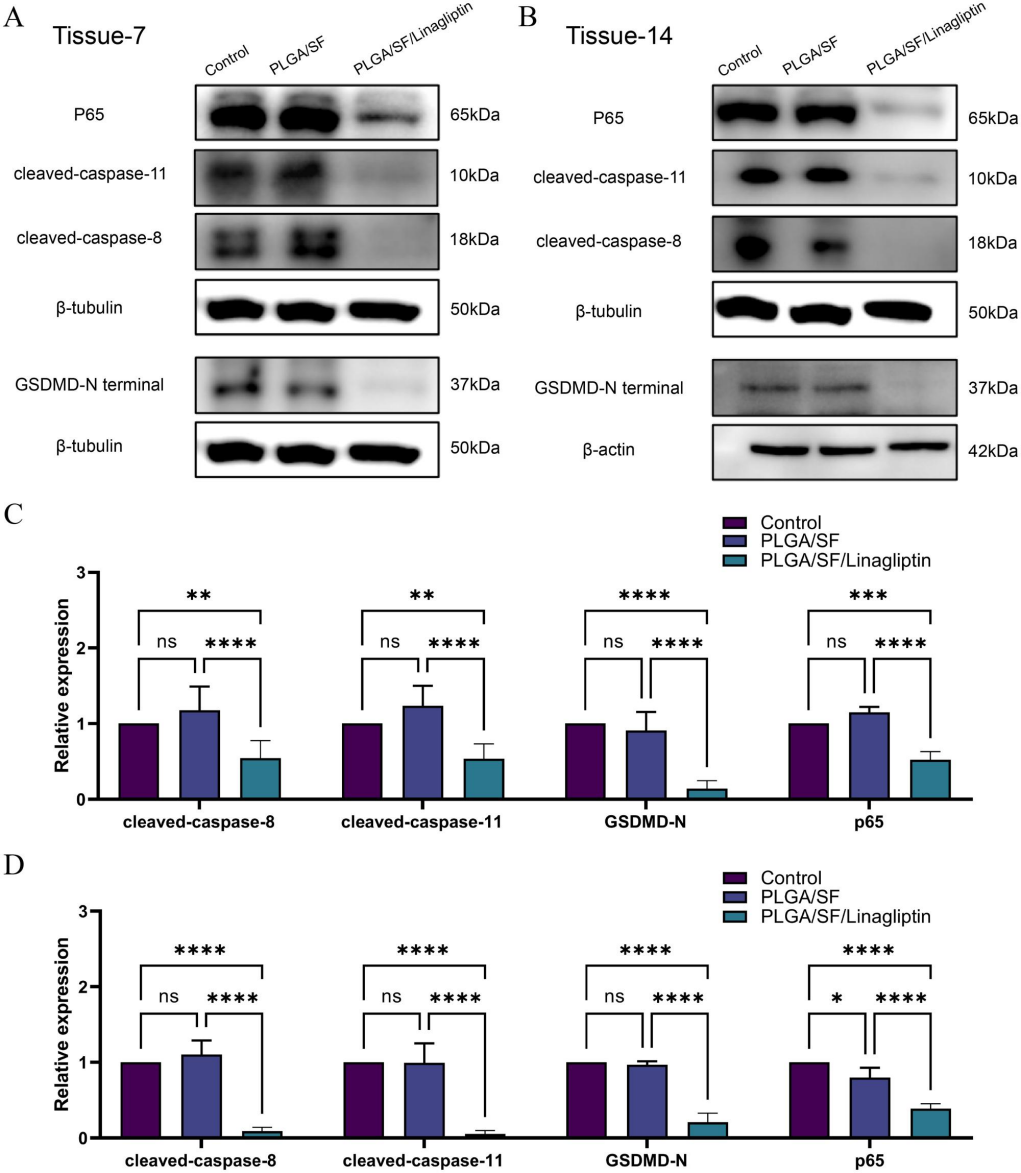
Pyroptosis protein expression in wound samples from different groups of animals. (**A**) Pyroptosis protein expression in different groups of experimental animals at 7 days. (**B**) Pyroptosis protein expression in different groups of experimental animals at 14 days. (**C**) The analysis of pyroptosis protein expression in different groups of experimental animals at 7 days. (**D**) The analysis of pyroptosis protein expression in different groups of experimental animals at 14 days. ns: *P* > 0.05, *: *P* ≤ 0.05, **: *P* ≤ 0.01, ***: *P* ≤ 0.001, ****: *P* ≤ 0.0001.

To further verify the above ideas, the expression of pyroptosis pathway-related proteins in animal tissue samples was assessed by immunoblotting assay. In the 7-day animal tissue samples, the protein expression of GSDMD-N, cleaved-caspase-11, cleaved-caspase-8 and P65 was significantly higher in the Control and PLGA/SF groups. Moreover, the expression of the above proteins was significantly lower in the PLGA/SF/Linagliptin group (Figure 7A and C). These findings indicated a more active pyroptosis signal and high expression of related proteins in the wound tissues of the Control and PLGA/SF groups. In the 14-day animal tissue samples, the protein expression of GSDMD-N, cleaved-caspase-11, cleaved-caspase-8 and P65 remained higher in the Control and PLGA/SF groups, indicating that the pyroptosis pathway was still persistently activated in the wound tissues of these two groups. In contrast, due to the slow release of linagliptin from the PLGA/SF/Linagliptin wound matrix-inducing membrane, the activation of the pyroptosis pathway was effectively inhibited, maintaining low expression of its related proteins (Figure 7B and D).

## Discussion

The impaired healing of chronic diabetic wounds is mainly attributed to the complex pathological microenvironment, particularly the persistent inflammation, excessive oxidative stress and impaired angiogenesis. To promote the wound healing, current research has shifted from passive physical coverings to smart biomaterials capable of microenvironmental responsiveness and precise immunomodulation [21]. As recently demonstrated by Xue *et al*. [22], using functionalized carriers (e.g. polysaccharide micelles) to overcome the delivery hurdles of hydrophobic drugs is inspiring and combining them with bioactive matrices like silk fibroin, can effectively drive macrophages to switch from a pro-inflammatory M1 phenotype to a regenerative M2 phenotype, thereby breaking the chronic inflammation. This strategy coincides with the insights proposed by Lei *et al*. [23] in their review, which argues that advanced biomaterials serve not only as drug reservoirs but as critical platforms for maintaining the stability of bioactive factors and achieving spatiotemporally controlled release.

Inspired by these pioneering studies, we engineered a Linagliptin/PLGA/SF wound matrix-induced membrane to regulate the wound microenvironment. What’s more, distinct from the research that focus on NF-κB pathways or canonical pyroptosis pathway in previous works, our study explores the noncanonical pyroptosis pathway. We aimed to demonstrate that linagliptin inhibits noncanonical pyroptosis in cells, and that a PLGA/SF based film, serving as a drug-loaded matrix, effectively delivers the drug to regulate inflammation and promote wound healing, which exhibits the consistent effect in both *in vitro* and *in vivo* experiments.

To determine Linagliptin’s cytotoxicity level, different concentrations of its solution were used during cell culture, and four key cells in wound healing were used [24, 25]. Results showed that linagliptin caused no significant toxicity to the four types of cells in the experimental group. Next, cobalt chloride with glucose solution was added to the culture medium to simulate the cellular microenvironment of diabetic wounds [5, 26, 27], and linagliptin treatment enhanced macrophage viability. The above results revealed that a certain concentration of linagliptin (6–18 μM) had no significant toxic effect on the major cells involved in wound healing. And under the high-glucose/hypoxic (HG/H) conditions, linagliptin (6–18 μM) was found to increase macrophage vitality, indicating the feasibility of drug intervention for diabetic wound treatment.

In diabetic wounds, pathological inflammation drives the excessive release of inflammatory mediators, significantly inhibiting the functions of endothelial cells and fibroblasts as well as keratinocytes, resulting in impaired wound angiogenesis, slow tissue remodeling and epithelialization malfunction [28–30]. Macrophages are the primary drivers of inflammation, and the diverse factors they secrete serve as critical roles in regulating the function of wound-healing-related cells. Therefore, regulating macrophage secretion to reduce excessive inflammatory mediator and enhance pro-migratory factor expression represents a promising strategy to promote wound healing. Our results indicate that in the high glucose and low oxygen environment of diabetic wounds, linagliptin (6–18 μM) regulates the paracrine action of macrophages (BMDM), thereby promoting the migration of fibroblasts and endothelial cells.

P65 is one of the key proteins of the NF-κB signaling pathway. Its activation forms a dimer that can function as a transcription factor in the nucleus to initiate the expression of a variety of genes. Many studies have shown that p65 mediates the increased expression of multiple inflammatory factors, including IL-1β, IL-6 and TNF-α, playing an important role in the pathogenesis of chronic inflammatory diseases. Moreover, chronic inflammation can be effectively inhibited by regulating p65 signaling [31–33]. Previous studies have shown that linagliptin can inhibit the NF-κB signaling pathway by suppressing the activity of the DPP4 molecule, thereby reducing the release of inflammatory factors [32, 34, 35]. Our results again verified the inhibitory effect of linagliptin on LPS-induced p65-related signaling pathway, indicating its potential to inhibit inflammatory signaling expression. In diabetic wounds, the direct anti-inflammatory effect of topical drugs can effectively promote healing.

A mixture of PLGA and silk fibroin was utilized to prepare the wound matrix-induced membrane. On the one hand, PLGA provided the material with suitable mechanical strength and slow-release properties. On the other hand, silk fibroin improved the biocompatibility and hydrophilicity of the material. Based on the results of Yan Peng et al., some adjustments were made to the ratio of these two materials [36–38]. Due to the strong hydrophilicity of SF proteins, both types of films showed relatively good hydrophilicity. The hydrophilic contact angle was less than 90° at the 1 s and 30 s time points of the contact between the water and the material. The bigger contact angle of the drug-loaded material films was due to the addition of linagliptin, whose hydrophobic nature alters the hydrophilicity of the copolymer. Regarding drug release, since diabetic wounds often persist in the inflammatory phase, sustained intervention against pathological inflammation within tissues is crucial. Our drug-loaded material exhibits excellent sustained-release properties and hydrophilicity, enabling continuous and stable release of linagliptin over two weeks, which can promote the transition of the wound to proliferative and remodeling phases. Specifically, the drug was released into PBS solution at a faster rate in the first 3 days of the experiment, referring to previous studies [36], this property may be attributed to the different hydrophilicity and solubility of the two polymers, PLGA and SF. In terms of mechanical strength, the incorporation of linagliptin enhances the mechanical properties of the fiber membranes and weakens their elastic deformation ability, this phenomenon may arise from the drug’s polar groups, which impede the mobility of molecular chains, reducing the extensibility of the membrane. According to a previous study, the stress of human skin ranges from 2.5 to 16.0 MPa, indicating that our prepared PLGA/SF/Linagliptin wound matrix-induced membrane can meet this stress requirement [36]. Our drug-loaded material exhibits sufficient strength for human skin without excessive deformation and material fracture. However, when applied to high-tension or highly mobile areas (e.g. joint surfaces), other high-strength materials may be required for reinforcement. Regarding biocompatibility, the extracts of two types of membranes did not show obvious toxic effects on the cells involved in wound healing or exhibit inhibitory effects on cell adhesion, demonstrating promising potential as biomaterials. The above results demonstrate that our prepared PLGA/SF/Linagliptin wound matrix-induced membrane has a more stable drug slow release, good hydrophilicity, good biocompatibility, can adapt to the mechanical properties of skin tissues, and can function as a wound dressing.

Previous studies have revealed that the key effectors of the pyroptosis pathway are the gasdermins. The key proteins in the pyroptosis pathway mediated by the GSDMD, it presented in both the classical and nonclassical pyroptosis pathways, include NLRP3, Caspase-1, Caspase-4/5/11, caspase-8, GSDMD and IL-1β [6, 26, 39]. As one of the classical pathways of inflammation, NF-kB can mediate the onset of pyroptosis and increased levels of NF-kB (p65) nucleation can effectively activate both classical and nonclassical pyroptosis pathways [6, 40, 41]. Other studies have demonstrated that in rotenone-induced rat models of Parkinson’s disease, linagliptin downregulate the canonical pyroptosis pathway to slow disease progression [42]. Furthermore, some researchers found that the absence of ASC protein expression in the classical pyroptosis pathway in Raw 264.7 prevented the assembly of effector inflammatory vesicles to activate Caspase-1 [9, 43]. Although a key component of the classical pathway (ASC inflammasomes) was missing in Raw 264.7 cells, previous studies have shown that caspase-4/5, their immediate homologue was CASP11 in mice and caspase-8 activate the GSDMD-induced nonclassical pyroptosis pathway and promote IL-1β maturation and release [44–49]. Therefore, our study aimed to explore the functional role of linagliptin in the noncanonical pyroptosis pathway. Specifically, pyroptosis is characterized by distinct morphological and plasma membrane alterations, including cellular swelling, collapse and the appearance of irregular vesicles shows a bubble-like protrusions and membrane pores [50, 51]. These changes result from oligomerization of the N-terminal domain of gasdermin proteins, forming plasma membrane pores that facilitate cytoplasmic leakage and cell lysis—features observable via scanning electron microscopy (SEM).

In our research, under the high-glucose/hypoxic (HG/H) conditions, expression of the GSDMD-N terminal was significantly elevated. Concurrently, electron microscopy revealed the characteristic pore formation in cell membrane, accompanied by cellular shrinkage and rupture. The significant alterations in the expression of proteins and cellular phenotypes indicated the activation of pyroptosis. Following linagliptin intervention, expression of GSDMD-N terminal was significantly downregulated and cellular morphology improved. These results demonstrate the drug’s capacity to inhibit macrophage pyroptosis. Previous studies have revealed the absence of inflammasomes required for the classical pyroptosis pathway in Raw 264.7 cells. Therefore, linagliptin likely exerts its effects via the nonclassical pyroptotic pathway. In noncanonical pyroptosis, both cleaved-caspase-8 and cleaved-caspase-11 can cleave GSDMD to release its active moiety. Our results showed that in the experimental group, the expression changes in cleaved-caspase-8 and cleaved-caspase-11 were consistent with those of GSDMD-N terminal, confirming our hypothesis. Previous studies have revealed that the noncanonical pyroptosis pathway contributes to inflammation in diabetic wounds. This suggests the potential of linagliptin in suppressing pathological inflammation of this kind of wounds.

Collectively, our results demonstrate that the HG/H microenvironment promotes macrophage pyroptosis. Linagliptin counteracts this effect by downregulating key protein expression within the noncanonical pyroptosis pathway, thereby inhibiting macrophage pyroptosis.

M2 macrophages, highly express CD163 in the cell surface, can suppress inflammation and promoting tissue repair. In diabetic wounds, the transition from M1 to M2 macrophages marks the shift into the reparative phase. These macrophages exert their effects through the secretion of anti-inflammatory factors (e.g. IL-4, IL-10) and pro-angiogenic factors. Consequently, high expression of CD163 indicates an abundance of M2 macrophages, suppressed inflammation and active tissue regeneration [52, 53]. Myeloperoxidase (MPO) is a critical marker of inflammation and neutrophil activation. Elevated MPO expression in tissues indicates active inflammation and oxidative stress. These results indicated that the drug-loaded material could inhibit the excessive inflammatory response, promote macrophage polarization to M2 type and the staining results of α-SMA and CD31 indicate that drug-loaded materials promote angiogenesis during the inflammatory phase of wound healing. On Day 14, the PLGA/SF/Linagliptin group demonstrated lower expression of CD163 and higher expression of Col-III collagen. The inflammatory cells in the wound tissue of this group subsided, initiating the process of tissue remodeling [54, 55].

In summary, the PLGA/SF/Linagliptin film can promote macrophage polarization to the M2 type, inhibit persistent wound inflammation and promote microangiogenesis in the early stage of diabetic wound healing, thus, regulating the wound microenvironment and accelerating tissue regeneration and wound healing.

Regarding *in vivo* experiments, in the PLGA/SF/Linagliptin group, upregulated genes include Tgfb2, Fgf3, Wnt3a, Tgfa and Areg. These genes accelerated wound re-epithelialization by promoting epidermal stem cell activation, keratinocyte migration and proliferation. Specifically, Tgfa, Areg and Ereg activate the MAPK pathway to promote keratinocyte migration; Fgf and Wnt signaling enhance cell proliferation and extracellular matrix remodeling; Krt16 and Tp63 maintain epithelial integrity. In this group, down-expressed genes include GSDMD, the interleukin family, Toll-like receptor family and caspase family. These genes are correlated with inflammation in the wound, promote sustaining inflammation and pyroptosis. The results of sequencing data indicated that compared with the control group and the PLGA/SF group, in the PLGA/SF/Linagliptin group, upregulated genes were enriched in biological processes such as re-epithelialization and cell-cell junction, whereas downregulated genes showed enrichment in inflammatory processes and pathways which served as crucial roles in promoting pathological inflammatory responses in wound tissues [56–58]. These results indicate that the drug-loaded material promoted the formation of skin barrier, suppressed multiple inflammation-related signaling pathways to promote wound healing process.

In summary, all the transcriptome sequencing analysis results showed that the PLGA/SF/Linagliptin group suppressed the expression of signaling pathways related to inflammation and pyroptosis and enhanced the expression of signaling pathways related to angiogenesis and tissue regeneration compared with the control and PLGA/SF groups. This demonstrated that linagliptin inhibits persistent wound inflammation, promotes tissue regeneration and accelerates diabetic wound healing by regulating these genes and signaling pathways.

In the PLGA/SF/Linagliptin group, linagliptin was released into the wound tissues during the healing process, which significantly suppressed the expression of proteins related to the pyroptosis pathway. This difference in expression was also consistent with the RNA-seq results described above. *In vitro* experiments demonstrated that linagliptin inhibits the expression of key proteins correlated with the noncanonical pyroptosis pathway in wound tissue. As previously described, P65 serves as a key protein of inflammation that activates the pyroptosis pathway. Among the three groups, the drug-loaded material group exhibited lower P65 levels than the other two groups, indicating that the sustained-release linagliptin from the drug-loaded material inhibits wound inflammation, consistent with the *in vitro* findings. Similarly, GSDMD-N terminal serves as an executor protein of pyroptosis that can be activated by cleaved-caspase-11 and cleaved-caspase-8 and its expression levels reflects pyroptosis activity. Across all three groups, expression differences in these three proteins were consistent with P65 patterns, indicating that the drug inhibited noncanonical pyroptosis in wound tissues. Given that our results *in vitro* have demonstrated that linagliptin modulates the noncanonical pyroptosis pathway to inhibit macrophage pyroptosis, suggesting this mechanism may be verified in our *in vivo* experiments.

Above all, the PLGA/SF/Linagliptin wound matrix-inducing membranes could inhibit the continuously activated pyroptosis pathway in wound tissues and regulate the inflammatory state of wounds through the sustained and stable release of linagliptin. We think that under inflammatory conditions, DPP4 engages surface receptors like CD32b to directly drive NF-κB activation. However, linagliptin disrupts this specific signaling axis, a conclusion verified by our results that nuclear P65 levels in macrophages reduced significantly with treatment. This blockage is critical because the transcription of caspase-11 (the key trigger for noncanonical pyroptosis) is dependent on nuclear NF-κB. By preventing P65 from the ‘priming stage’ of noncanonical pyroptosis pathway, linagliptin suppresses the supply of caspase-11, which in turn effects downstream GSDMD cleavage and caspase-8 activation.

In this study, we did not employ gene knockout or knockdown animal models. We recognize that the intracellular signaling pathways have not been fully experimentally verified and the specific molecular function of DPP4 was not explored in depth. Our study prioritized the translational potential and therapeutic efficacy of the functionalized biomaterial. And we position genetic validation as a necessary direction for our future research.

## Conclusion

In this study, linagliptin was found to inhibit macrophage pyroptosis by downregulating the expression of key proteins in the nonclassical pyroptosis pathway, thereby improving cellular function and modulating cellular inflammation. A wound matrix-inducing membrane with slow linagliptin release was prepared, promoting chronic wound healing by modulating macrophage pyroptosis and inducing changes in the wound matrix microenvironment.

## Supplementary Material

rbag048_Supplementary_Data

## Data Availability

The RNA sequencing data have been deposited in the NCBI database under the Sequence Read Archive (SRA) accession number PRJNA1335785. Other data that supports the findings of this study are available from the corresponding author upon reasonable request.
