# Supplementary material for: PLGA/SF/linagliptin wound matrix-induced membrane promotes diabetic wounds healing by inhibiting macrophage pyroptosis
Source: Regen Biomater. 2026 May 8;13:rbag048. doi: 10.1093/rb/rbag048 (PMC13283444; doi:10.1093/rb/rbag048)
Supplement: rbag048_Supplementary_Data [file rbag048_supplementary_data.zip › Supplementary_Information_for Regenerative_Biomaterial.docx]

**Supplementary Information (SI)**

**PLGA/SF/linagliptin wound matrix-induced membrane promotes diabetic wounds healing by inhibiting macrophage pyroptosis**

**This file includes:**

**Supplementary figures**

1. Under HG/H conditions, we treated Raw 264.7 cells with different concentrations of linagliptin, then conducted WB experiments.


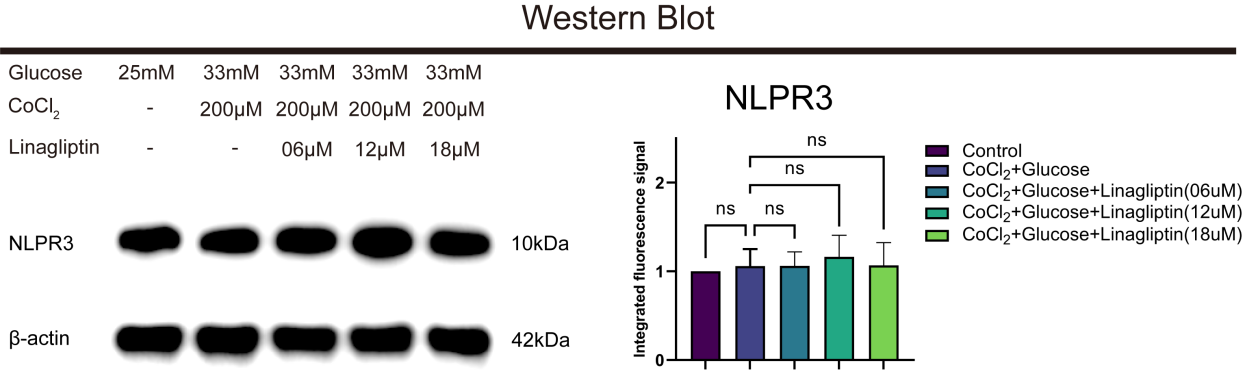


**Supplementary Figure 1** After treating Raw 264.7 cells with different concentrations of linagliptin for 48 hours, the expression of NLRP3 was measured using immunofluorescence experiments.

2. The PLGA/SF ratio was screened preliminarily based on a reported formulation (referenced as a guide from https://doi.org/10.1016/j.ijbiomac.2021.04.021). Specifically, three formulations of drug-loaded films were fabricated: Film a: PLGA 1.0 g, SF 0.1 g, Linagliptin 6.5 mg, Film b: PLGA 1.0 g, SF 0.3 g, Linagliptin 6.5 mg, and Film c: PLGA 1.0 g, SF 0.5 g, Linagliptin 6.5 mg. Subsequent material characterization revealed that Film a displayed poor hydrophilicity, as indicated by its water contact angle [Supplementary Figure 2A], while Film c showed inadequate tensile strength and elastic deformation in stress-strain measurements [Supplementary Figure 2B]. Therefore, based on these findings, the composition of Film b was ultimately adopted for all further experiments.


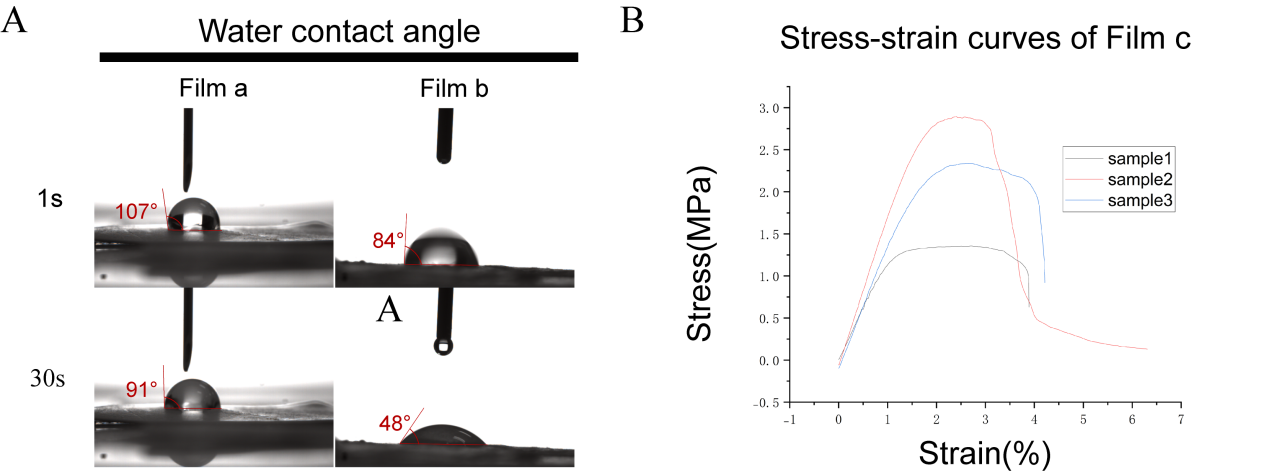


**Supplementary Figure 2** (A) The water contact angles of “Flim a” and “Film b”. (B) The stress-strain curves of samples from “Flim c”.

3. Since the wound dressing is designed as a non-implantable material, we performed a long-term in vitro degradation test as an alternative to simulate the degradation curve [Supplementary Figure 3].


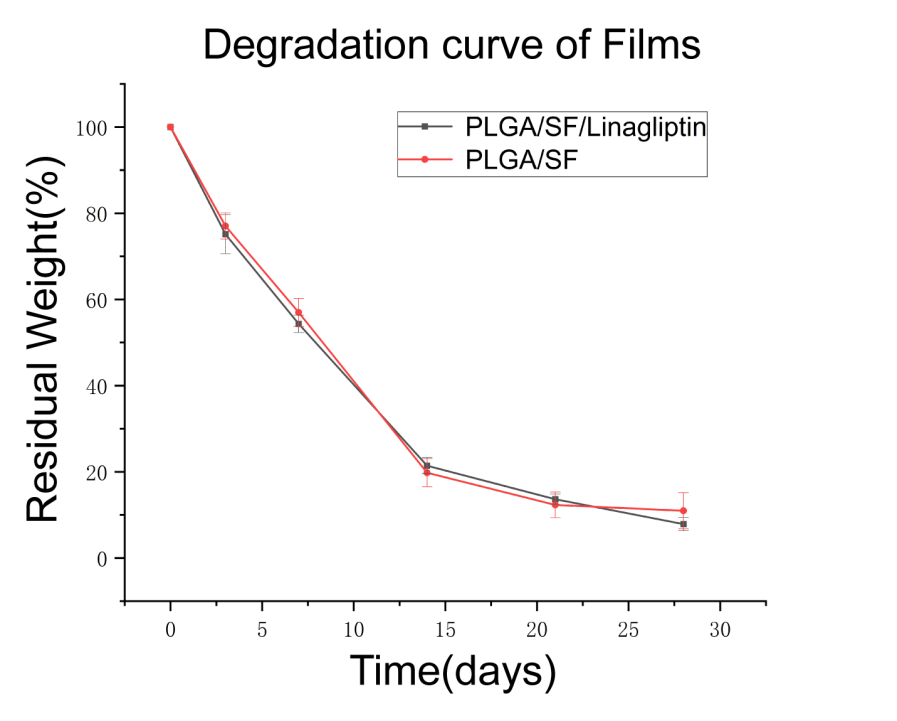


**Supplementary Figure 3** The degradation curve of PLGA/SF and PLGA/SF/Linagliptin films.

4. In our preliminary experiments, three distinct drug-loaded films were fabricated: Film-low: PLGA 1.0 g, SF 0.3 g, Linagliptin 3.25 mg, Film-medium: PLGA 1.0 g, SF 0.3 g, Linagliptin 6.5 mg, and Film-high: PLGA 1.0 g, SF 0.3 g, Linagliptin 13 mg. We conducted some experiments to select the suitable film: the CCK-8 assay showed that Film-high was cytotoxic [Supplementary Figure 4C] and released the drug too rapidly to provide the sustained effect [Figure 2A of the manuscript]. On the other hand, Film-low failed to inhibit macrophage pyroptosis [Supplementary Figure 4A-B]. Consequently, we selected Film-medium as the suitable film for the subsequent experiments.


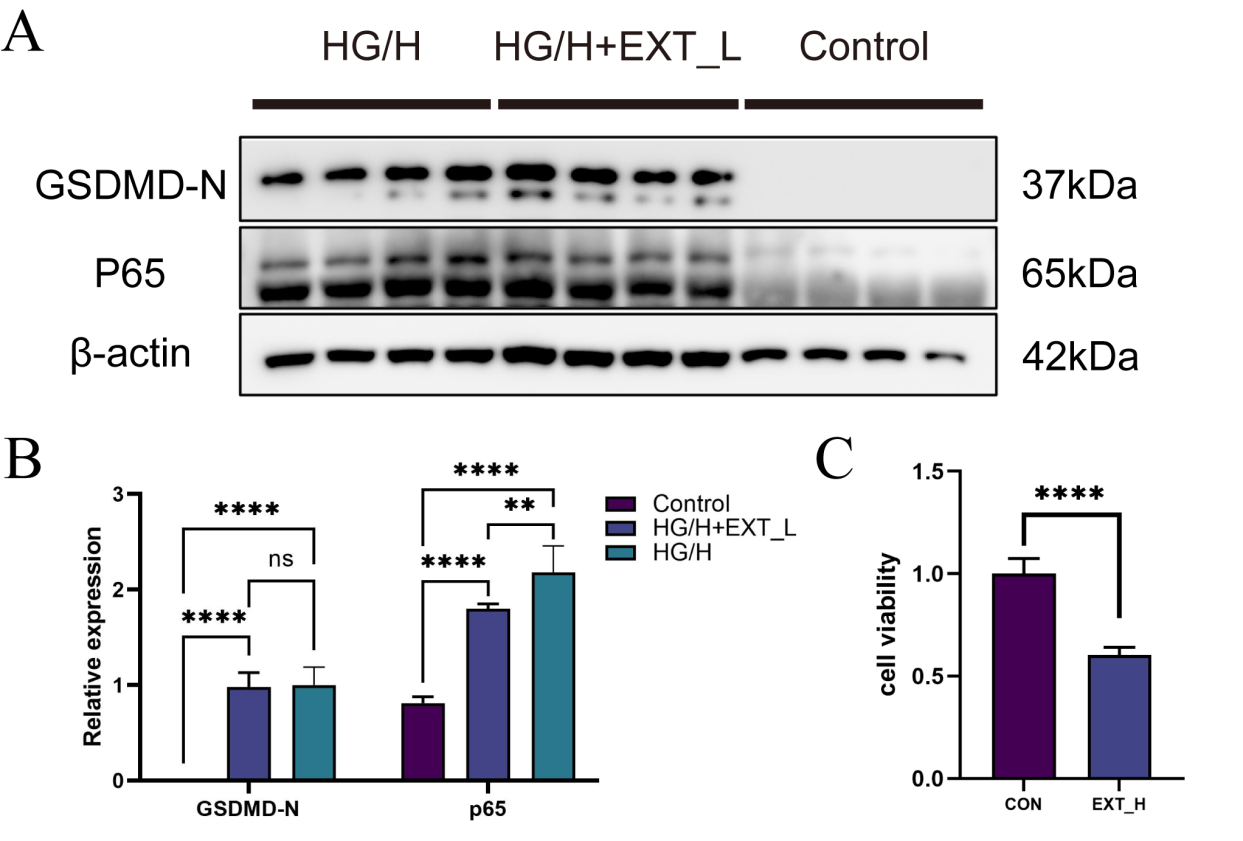


**Supplementary Figure 4** (A) The WB of different treatments(HG/H: high glucose and hypoxia; EXT_L: extract material of Film-low). (B) The analysis of the WB. (C) The CCK8 assey of control group and EXT_H group (EXT_H: extract material of Film-high)

**Supplementary table**

| **Company** | **Product Name** | **Catalog Number** |
| --- | --- | --- |
| MedChemExpress [MCE], USA | Linagliptin | HY-10284 |
| Nanjing JG Chemicals Co., Ltd., China | Silk fibroin | S36042 |
| Shanghai Jinhong Science & Technology Co., Ltd., China | PLGA | DG-50DLGH080 |
| Millipore, USA | PVDF membrane | E802-01 |
| Solarbio Science & Technology Co., Ltd., Beijing, China | DAPI solution | C0065 |
| Biosharp, Beijing, China | SDS-PAGE sample loading buffer | BL502B |
| Beyotime Biotechnology, Shanghai, China | RIPA lysis buffer | P0013B |
| Biosharp, Beijing, China | CCK-8 kit | BS350B |
| ABclonal Technology, Wuhan, China | IL-1 beta ELISA kit | RK00006 |
| Beyotime Biotechnology, Shanghai, China | BCA Protein Assay Kit | P0012 |
| Wanlei Life Sciences, Shenyang, China | Anti-Caspase-8 p18 antibody | WL00659 |
| UpingBio Technology Co., Ltd., Shenzhen, China | Anti-Caspase-11 p10 antibody | YP-mAb-00019 |
| ABclonal Technology, Wuhan, China | Anti-GSDMD (Full length and N terminal) antibody | A24476 |
| Beyotime Biotechnology, Shanghai, China | Anti-NF-κB p65 antibody | AF0246 |
| ABclonal Technology, Wuhan, China | Anti-beta-actin antibody | AC026 |
| ABclonal Technology, Wuhan, China | Anti-beta-tubulin antibody | A12289 |
| ABclonal Technology, Wuhan, China | Anti-GAPDH antibody | A19056 |
| Proteintech Group, Wuhan, China | Anti-Histone H4 antibody | 16047-1-AP |
| ABclonal Technology, Wuhan, China | HRP-conjugated Goat Anti-Mouse IgG (H+L) | AS003 |
| ABclonal Technology, Wuhan, China | HRP-conjugated Goat Anti-Rabbit IgG (H+L) | AS014 |

**Supplementary table 1** The information of key materials in the research.
