# Supplementary figures and images for: PLGA/SF/linagliptin wound matrix-induced membrane promotes diabetic wounds healing by inhibiting macrophage pyroptosis
Source: Regen Biomater. 2026 May 8;13:rbag048. doi: 10.1093/rb/rbag048 (PMC13283444; doi:10.1093/rb/rbag048)

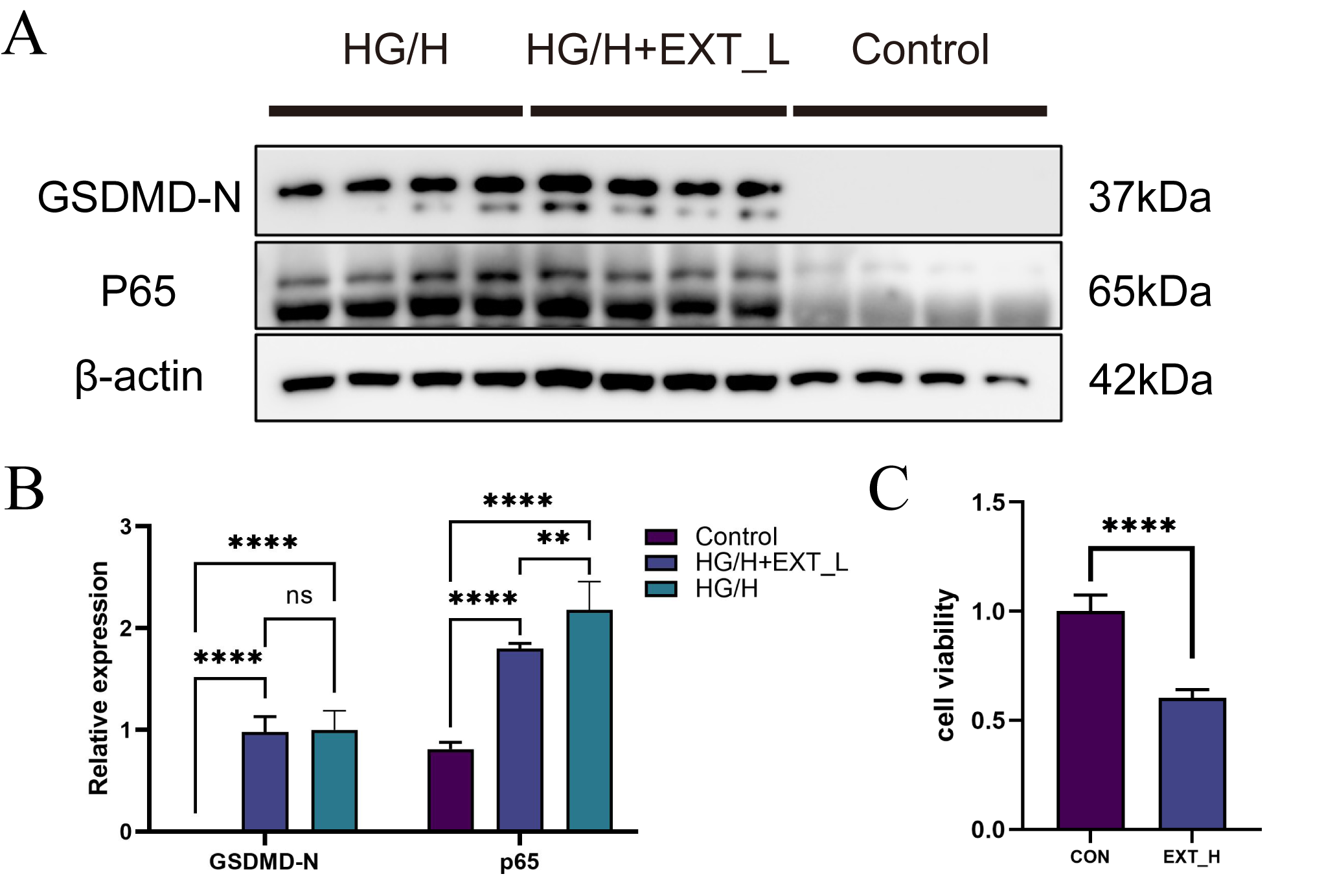

Supplement: rbag048_Supplementary_Data [file rbag048_supplementary_data.zip › Supplement_Figure_4.png]

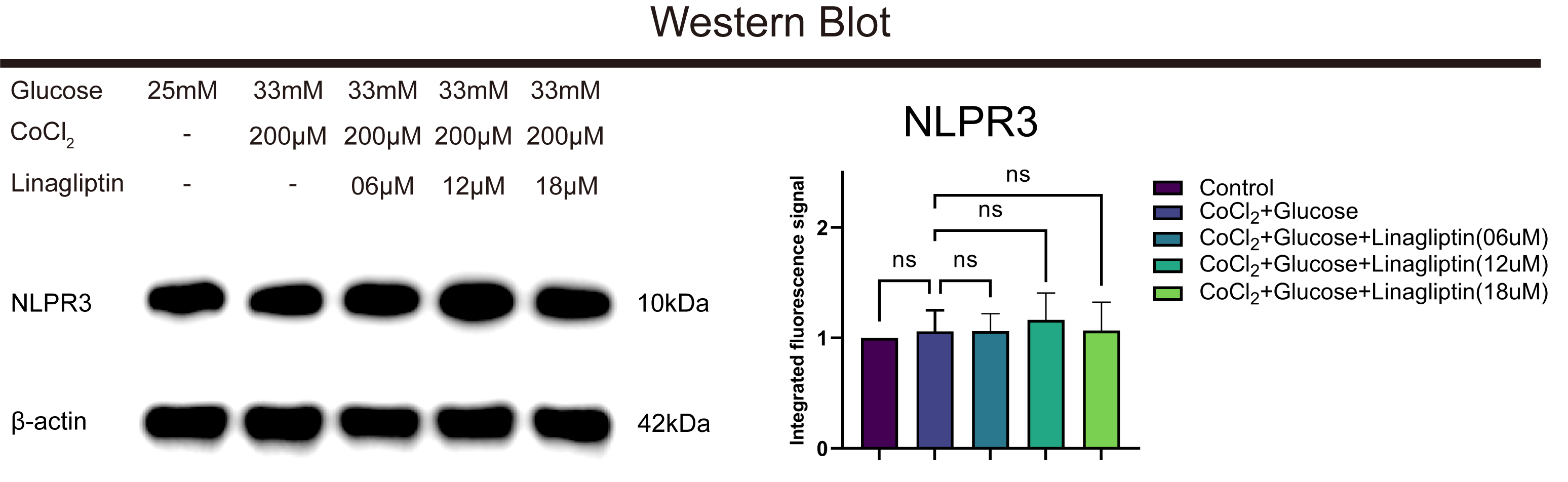

Supplement: rbag048_Supplementary_Data [file rbag048_supplementary_data.zip › Supplement_Figure_1.png]

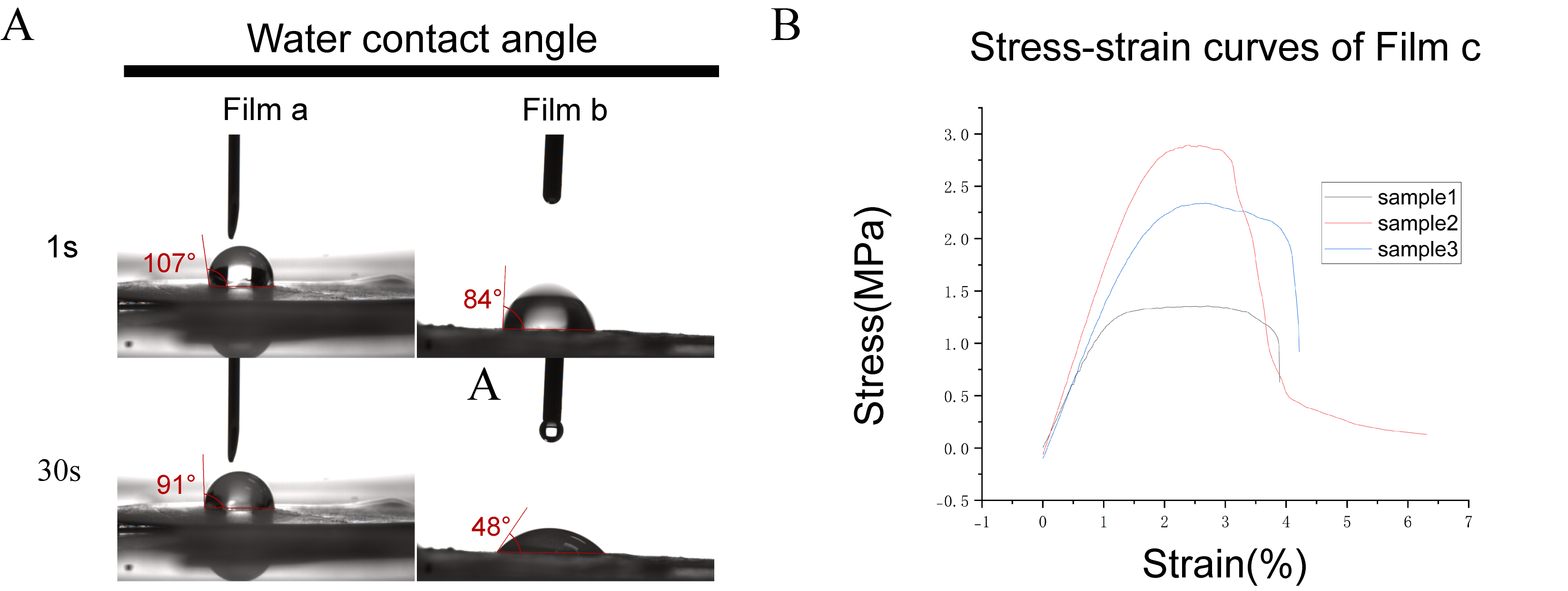

Supplement: rbag048_Supplementary_Data [file rbag048_supplementary_data.zip › Supplement_Figure_2.png]

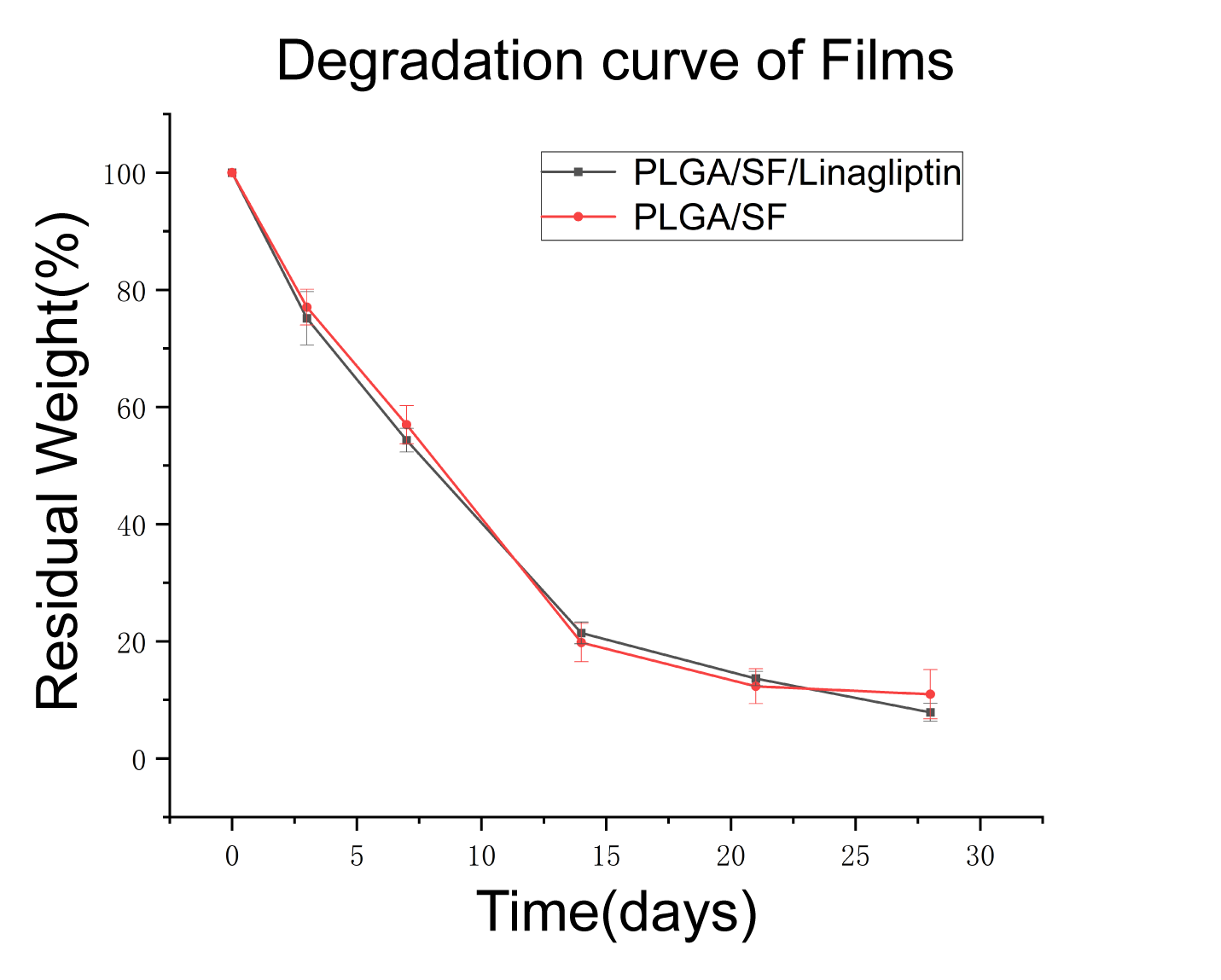

Supplement: rbag048_Supplementary_Data [file rbag048_supplementary_data.zip › Supplement_Figure_3.png]
