# Supplementary material for: PLGA/SF/linagliptin wound matrix-induced membrane promotes diabetic wounds healing by inhibiting macrophage pyroptosis
Source: Regen Biomater. 2026 May 8;13:rbag048. doi: 10.1093/rb/rbag048 (PMC13283444; doi:10.1093/rb/rbag048)
Supplement: rbag048_Supplementary_Data [file rbag048_supplementary_data.zip › Response_to_reviewers.docx]

Response to reviewers

I’m very grateful for your letter and for the reviewers’ comments concerning our manuscript entitled “PLGA/SF/linagliptin wound matrix-induced membrane promotes diabetic wounds healing by inhibiting macrophage pyroptosis” (ID: RB-2025-630). Those comments are all valuable and very helpful for revising and improving our paper, as well as the important guiding significance to our researches. We have studied comments carefully and have made correction which we hope meet with approval.

The main corrections in the paper and the responds to the reviewer’s comments are as flowing:

***Reviewer: 1***

*Comment 1. What is the rationale for selecting the mixing ratio of PLGA to SF (1:0.3)? It is recommended to supplement relevant materials or illustrate the rationality of this ratio through preliminary experiments with different ratios .*

The PLGA/SF ratio was screened preliminarily based on a reported formulation (referenced as a guide from https://doi.org/10.1016/j.ijbiomac.2021.04.021). Specifically, three formulations of drug-loaded films were fabricated: Film a: PLGA 1.0 g, SF 0.1 g, Linagliptin 6.5 mg, Film b: PLGA 1.0 g, SF 0.3 g, Linagliptin 6.5 mg, and Film c: PLGA 1.0 g, SF 0.5 g, Linagliptin 6.5 mg. Subsequent material characterization revealed that Film a displayed poor hydrophilicity, as indicated by its water contact angle [Figure R1A], while Film c showed inadequate tensile strength and elastic deformation in stress-strain measurements [Figure R1B]. Therefore, based on these findings, the composition of Film b was ultimately adopted for all further experiments.

Figure R1.(A) The water contact angles of “Flim a” and “Film b”. (B) The stress-strain curves of samples from “Flim c”.

*Comment 2. What is the specific mechanism by which linagliptin acts on the non-canonical pyroptosis pathway? It is recommended to improve the relevant mechanistic chain.*

We thank the reviewer for this valuable insight sincerely. We agree that the specific mechanism by which linagliptin acts on the non-canonical pyroptosis pathway is very important for the study.

Our results and recent findings (e.g., Tang et al., Mol. Ther. 2021) suggest that linagliptin inhibits non-canonical pyroptosis by interrupting DPP4-dependent upstream signaling (DPP4/CD32b/NF-kB axis). It prevents NF-κB from the "priming stage" of non-canonical pyroptosis pathway.

We think that under inflammatory conditions, DPP4 engages surface receptors like CD32b to directly drive NF-κB activation. However, linagliptin disrupts this specific signaling axis, a conclusion verified by our results that nuclear P65 levels in macrophages reduced significantly with treatment. This blockage is critical because the transcription of caspase-11 (the key trigger for non-canonical pyroptosis) is dependent on nuclear NF-κB. By preventing P65 from the "priming stage" of non-canonical pyroptosis pathway, linagliptin surpresses the supply of caspase-11, which in turn effects downstream GSDMD cleavage and caspase-8 activation.

In short, Linagliptin → Inhibits DPP4 signaling (e.g., DPP4/CD32b) → Inhibits NF-κB nuclear entry → Suppresses caspase-11 → Reduces caspase-8 activation and GSDMD cleavage.

References:

1. Neural Regen Res. 2024 Jul 1;19(7):1608-1617. doi: 10.4103/1673-5374.386490. Epub 2023 Nov 8. PMID: 38051906; PMCID: PMC10883521.
2. Immunity. 2018 Jul 17;49(1):42-55.e6. doi: 10.1016/j.immuni.2018.06.011. PMID: 30021146; PMCID: PMC6064639.
3. Tissue Cell. 2025 Apr;93:102672. doi: 10.1016/j.tice.2024.102672. Epub 2024 Dec 10. PMID: 39721419.
4. Nat Rev Drug Discov. 2021 May;20(5):384-405. doi: 10.1038/s41573-021-00154-z. Epub 2021 Mar 10. PMID: 33692549; PMCID: PMC7944254.
5. Mol Aspects Med. 2020 Dec;76:100924. doi: 10.1016/j.mam.2020.100924. Epub 2020 Nov 11. PMID: 33187725; PMCID: PMC7808250.
6. Autophagy. 2019 May;15(5):843-870. doi: 10.1080/15548627.2019.1569913. Epub 2019 Jan 27. Erratum in: Autophagy. 2023 Jan;19(1):374-375. doi: 10.1080/15548627.2022.2074778. PMID: 30653446; PMCID: PMC6526809.
7. Mol Ther. 2021 Jan 6;29(1):365-375. doi: 10.1016/j.ymthe.2020.08.017. Epub 2020 Sep 5. PMID: 32956626; PMCID: PMC7790911.

*Comment 3. It is recommended to add data on the long-term degradation curve of the composite membrane in vivo, as well as detection of tissue inflammatory responses and drug residues, to supplement long-term biosafety data.*

We appreciate your valuable suggestions regarding the material's properties. Linagliptin is a widely used and relatively safe drug in clinical practice. Since the wound dressing is designed as a non-implantable material, conducting long-term in vivo degradation experiments poses significant challenges; therefore, we performed a long-term in vitro degradation test as an alternative to simulate the degradation curve (Figure R2A) and supplemented the hemolysis data to further validate biosafety (Figure R2B). Regarding tissue inflammation, our Myeloperoxidase (MPO) staining on Day 7 samples indicated that the drug-loaded materials supressed the inflammatory response (these data are exhibited in the Figure 5 of our manuscript). While accurately drug residues in tissue is difficult to test, we hope the in vitro drug release experiments in our manuscript serve as a valuable reference.

Figure R2.(A) The degradation curve of PLGA/SF and PLGA/SF/Linagliptin films. (B) The hemolysis test of the material extracts (EXT_1: the material extracts of PLGA/SF, EXT_2: the material extracts of PLGA/SF/Linagliptin).

*Comment 4. For Figure 5, it is suggested to supplement quantitative analysis of fluorescence intensity and statistical analysis of inter-group differences, avoiding over-reliance on qualitative observation alone.*

We fully agree with the suggestion that quantitative analysis is crucial. Therefore, we have conducted staining and statistical analysis on additional samples. The updated results, including statistical comparisons between groups, have been incorporated into the revised Figure 5 of the manuscript.To facilitate your review, we also display the results below (Figure R3A-E).

Figure R3. (A) CD163 immunofluorescence staining results at 7 and 14 days in different groups of experimental animals. (B) Immunofluorescence staining results for CD31 and α-SMA at 7 days in experimental animals from different groups. (C) Immunofluorescence staining results for MPO at 7 days in experimental animals from different groups. (D) Immunofluorescence staining results for Col III at 14 days in experimental animals from different groups. (E) The analysis of all markers

*Comment 5. For the same drug, the concentration required to achieve therapeutic effects varies with different administration routes. To clarify the optimal drug loading ratio and dose-effect relationship, it is recommended to supplement efficacy gradient experiments of composite membranes with different linagliptin concentrations to enhance experimental completeness.*

In our preliminary experiments, three distinct drug-loaded films were fabricated: Film-low: PLGA 1.0 g, SF 0.3 g, Linagliptin 3.25 mg, Film-medium: PLGA 1.0 g, SF 0.3 g, Linagliptin 6.5 mg, and Film-high: PLGA 1.0 g, SF 0.3 g, Linagliptin 13 mg. We conducted some experiments to select the suitable film: the CCK-8 assay showed that Film-high was cytotoxic (Figure R4C) and released the drug too rapidly to provide the sustained effect (Figure 2A of the manuscript). On the other hand, Film-low failed to inhibit macrophage pyroptosis (Figure R4A-B). Consequently, we selected Film-medium as the suitable film for the subsequent experiments.

Figure R4.(A) The WB of different treatments(HG/H: high glucose and hypoxia; EXT_L: extract material of Film-low). (B) The analysis of the WB. (C) The CCK8 assey of control group and EXT_H group (EXT_H: extract material of Film-high)

*Comment 6. In the discussion section, the comparative analysis with existing studies related to "pyroptosis regulation + wound healing" should be strengthened, and the innovations and limitations of this study should be objectively explained.*

We appreciate the reviewer’s insight, which prompted a necessary restructuring of our discussion section to better explain our findings. We have updated the manuscript to include the following conclusions:

Mechanism and innovation:

Pyroptosis, especially the classical pyroptosis pathway, is a hot topic in current research. By regulating pyroptosis pathways, researchers can intervene in various pathological inflammatory processes (e.g., diabetic wounds). Rather than following the classical NLRP3/Caspase-1 axis in current research, our study focus on the often-overlooked non-canonical pyroptosis pathway. In the specific microenvironment of high glucose and hypoxia (HG/H), we observed that our drug-loaded films serves as more than a physical barrier; it actively intervenes in the pyroptosis signaling pathway. By blocking P65 nuclear translocation, the drug-loaded films suppressed the downstream activation of Caspase-11 and Caspase-8, thereby inhibiting GSDMD cleavage and protecting cells. The interruption of the non-canonical pyroptosis pathway defines the primary innovation of our work. What’s more, this study is the first to repurpose linagliptin, an oral drug, for localized diabetic wound using a sustained-release material approach.

Objective limitations:

In this study, we did not employ gene knockout or knockdown animal models. We recognize that the intracellular signaling pathways have not been fully experimentally verified, and the specific molecular function of DPP4 was not explored in depth. Our study prioritized the translational potential and therapeutic efficacy of the functionalized biomaterial. We have established a logical chain through Western Blot, SEM, animal models and immunofluorescence and we position genetic validation as a necessary direction for our future research.

*Comment 7. Some previous works were suggested to read and discuss in this work, such as: Materials Today Bio,35,2025,102356; Materials & Design, 257,2025,114505; Biomater Res. 2025;29:0148.DOI:10.34133/bmr.0148；*

We thank the reviewer for recommending these high-impact references. After a thorough review, we found these works are scientific and rigorous, offering valuable insights that we have now integrated into our revised discussion section.

The recent study by Xue et al. in Materials Today Bio presents a "monitoring-treatment" microneedle system. Their approach, which combines real-time pH sensing with regulating NRF2 and NFκB pathways, sets a high standard for intelligent wound management. And their work in Materials & Design offers a sophisticated solution to the delivery of Ginkgolide B using polysaccharide micelle hydrogel. This strategy, enhancing bioavailability and effectively drive M2 macrophage polarization is similar with our design of using nanofiber films for the sustained delivery of Linagliptin. Furthermore, the review by Lei et al. in Biomaterials Research provides an overview of exosome-based therapeutics, reinforcing the critical roles of advanced biomaterials in modulating the microenvironment in complex wounds.

Inspired by these pioneering studies, we have revised our discussion to explain the potential of our Linagliptin-loaded nanofiber film to serve as functional dressings. As the advanced systems described in the recommended articles, our material functions as an active modulator of the wound microenvironment. While our study, focusing on the regulation of non-canonical pyroptosis axisl, remains aligned with these research which use the functional materials to disrupt the cycle of chronic inflammation and accelerate tissue regeneration. Drawing on these ideas, I believe our future study can be more comprehensive and in-depth. We have cited these references in the manuscript to reflect the rationality and relevance of our findings.

***Reviewer: 2***

*Comments to the Author*

1. *In the research, it is proved that linagliptin promotes diabetic wounds healing and inhibits macrophage pyroptosis, but the evidence showing linagliptin promotes diabetic wounds healing by inhibiting macrophage pyroptosis is not sufficient.*

We sincerely appreciate the reviewer's comment. We agree that showing linagliptin heals wounds specifically by blocking macrophage pyroptosis is challenging. In the revised manuscript, we have clarified our limitations and laid out future plans.

It's known that chronic inflammation makes diabetic wounds hard to heal and macrophages are the most crucial cells mediating the inflammation. So in our study, we focus on macrophages and its pyroptosis. Our study showed that linagliptin-loaded films actively alters this inflammatory phase and promotes wound healing. In vitro experiments, under high glucose and hypoxia conditions, linagliptin surpressed the non‑canonical pyroptosis pathway: the results exhibited the lower expression of nuclear P65, active-caspase‑11/8, and GSDMD N terminal. Importantly, what we saw in animals matched these findings — as wounds closed faster, inflammation subsided (MPO), and wound healing improved (more CD163 and Col III). This consistency across different levels strongly suggests that reducing macrophage pyroptosis is a key way our drug-loaded films helps repair.

We think that under inflammatory conditions, DPP4 engages surface receptors like CD32b to directly drive NF-κB activation. However, linagliptin disrupts this specific signaling axis, a conclusion verified by our results that nuclear P65 levels in macrophages reduced significantly with treatment. This blockage is critical because the transcription of caspase-11 (the key trigger for non-canonical pyroptosis) is dependent on nuclear NF-κB. By preventing P65 from the "priming stage" of non-canonical pyroptosis pathway, linagliptin surpresses the supply of caspase-11, which in turn effects downstream GSDMD cleavage and caspase-8 activation.

We acknowledge that without genetic knockout or rescue tests, we can’t exclude other contributing factors. We’ve added this point to the discussion as the limitation. And we plan to use gene‑edited models to directly explore the role of specific pathway chain mediated by the DPP4 molecule in cell signal transduction. Still, we believe our current work shows this biomaterial has potential to offer both mechanical barrier and regulate inflammation effectively for diabetic wounds.

*2. The methods of mice BMDM culture and the high glucose/hypoxic condition culture were not clearly shown in the manuscript.*

We fully appreciate your attention to the experimental conditions. In our study, to simulate the microenvironment of diabetic wounds, we treated mice BMDM with a combination of high glucose (HG) and cobalt chloride (CoCl₂). The concentrations were set at 200 μM for CoCl₂ and 33 mM for glucose. This formulation aims to induce cellular responses reliably, thereby providing a more physiologically relevant context for investigating diabetic wound healing mechanisms.

***Reviewer: 3***

*Comments to the Author*

*This manuscript describes a novel wound matrix-inducing membrane with slow linagliptin release that promotes chronic wound healing by modulating macrophage pyroptosis and changing the wound matrix microenvironment. The efficacy and mechanism of promoting diabetic wound healing were well demonstrated through in vitro and in vivo experiments. Overall, I believe this work is suitable for publication in Regenerative Biomaterials after minor revision.*

1. *For the characterization of PLGA/SF and PLGA/SF/Linagliptin wound matrix-induced membranes, it might be helpful to analyze the chemical structure of membranes using FTIR spectroscopy.*

We thank the reviewer for the suggestion to include FTIR data. In response, we have characterized the FTIR spectra for all components and films (updated in Figure 2 of the manuscript). To facilitate your review, we also display the results below (Figure R5).

Pure PLGA displayed characteristic absorption peaks at 2999 cm⁻¹, 2949 cm⁻¹, 1747 cm⁻¹, 1455 cm⁻¹, 1386 cm⁻¹, 1171 cm⁻¹, 1083 cm⁻¹, 867 cm⁻¹, and 749 cm⁻¹, all peaks are consistent with the linear polymeric structure of PLGA. Similarly, pure silk fibroin (SF) showed peaks at 3286 cm⁻¹, 3071 cm⁻¹, 2936 cm⁻¹, 1640 cm⁻¹, 1513 cm⁻¹, 1229 cm⁻¹, and 1061 cm⁻¹, which align closely with reference spectra reported for SF.

In the PLGA/SF membranes, each observed peak could be clearly attributed to either PLGA or SF, with no significant shift or disappearance of the characteristic PLGA absorptions. This indicates that no pronounced chemical interaction occurred between the two components during fabrication.

Notably, in the PLGA/SF/Linagliptin membranes, we did not detect distinct FTIR peaks corresponding to linagliptin. This is likely due to its relatively low weight in the membranes and the overlap of its characteristic bands with the strong absorption signals from PLGA.

Figure R5. The FTIR spectra of all components and membranes.

2. *It is commendable that the authors provided detailed experimental procedures. However, I suggest including product numbers of key materials (e.g., raw materials, kits, antibodies, PVDF membranes, etc.).*

We appreciate your attention to the product numbers of key materials. Here we provide these information for you.

Materials & Reagents:

Linagliptin: (Cat. No. HY-10284, MedChemExpress [MCE], USA)

Silk fibroin: (Cat. No. S36042, Nanjing JG Chemicals Co., Ltd., China)

PLGA: (Cat. No. DG-50DLGH080, Shanghai Jinhong Science & Technology Co., Ltd., China)

PVDF membrane: (Cat. No. E802-01, Millipore, USA)

DAPI solution: (Cat. No. C0065, Solarbio Science & Technology Co., Ltd., Beijing, China)

SDS-PAGE sample loading buffer: (Cat. No. BL502B, Biosharp, Beijing, China)

RIPA lysis buffer: (Cat. No. P0013B, Beyotime Biotechnology, Shanghai, China)

Kits:

CCK-8 kit: (Cat. No. BS350B, Biosharp, Beijing, China)

IL-1 beta ELISA kit: (Cat. No. RK00006, ABclonal Technology, Wuhan, China)

BCA Protein Assay Kit: (Cat. No. P0012, Beyotime Biotechnology, Shanghai, China)

Antibodies:

Anti-Caspase-8 p18 antibody: (Cat. No. WL00659, Wanlei Life Sciences, Shenyang, China)

Anti-Caspase-11 p10 antibody: (Cat. No. YP-mAb-00019, UpingBio Technology Co., Ltd., Shenzhen, China)

Anti-GSDMD (Full length and N terminal) antibody: (Cat. No. A24476, ABclonal Technology, Wuhan, China)

Anti-NF-κB p65 antibody: (Cat. No. AF0246, Beyotime Biotechnology, Shanghai, China)

Anti-beta-actin antibody: (Cat. No. AC026, ABclonal Technology, Wuhan, China)

Anti-beta-tubulin antibody: (Cat. No. A12289, ABclonal Technology, Wuhan, China)

Anti-GAPDH antibody: (Cat. No. A19056, ABclonal Technology, Wuhan, China)

Anti-Histone H4 antibody: (Cat. No. 16047-1-AP, Proteintech Group, Wuhan, China)

HRP-conjugated Goat Anti-Mouse IgG (H+L): (Cat. No. AS003, ABclonal Technology, Wuhan, China)

HRP-conjugated Goat Anti-Rabbit IgG (H+L): (Cat. No. AS014, ABclonal Technology, Wuhan, China)

We have revised these information for the “Materials” in manuscript.

3. *Even though the manuscript is well-written, some formatting issues need to be addressed. For example, in line 130, “15cm” should be “15 cm”; in line 226, “1×106cell/well” should be “1×106 cells/well”.*

We sincerely thank you for your meticulous review, which is helpful in improving the standardization of our manuscript. We have carefully corrected these formatting details in the revised text.

***Reviewer: 4***

*Comments to the Author*

*Diabetic wounds often exhibit delayed healing due to persistent inflammation, largely driven by macrophage pyroptosis, which results in excessive release of inflammatory mediators. In this study, the authors load linagliptin onto a PLGA/SF composite membrane prepared via electrospinning to modulate pathological inflammation and promote diabetic wound healing. While the work is meaningful, several issues require clarification and improvement.*

1. *The introduction provides a comprehensive overview of diabetes and related therapeutic mechanisms but lacks justification for the selection of PLGA and SF as the scaffold materials. The authors should supplement the background with a discussion of the advantages of PLGA and SF—such as biocompatibility, biodegradability, mechanical properties, and clinical relevance—and clearly explain why these materials and their preparation method were chosen over other clinically approved excipients. This addition will help readers better understand the rationale and significance of the study.*

We appreciate the reviewer’s comment regarding the rationale for selecting poly(lactic-co-glycolic acid) (PLGA) and Silk Fibroin (SF). The choice to prepare a composite scaffold from these two distinct materials was driven by the specific clinical demands of wound management.

PLGA offers adjustable biodegradability and strong mechanical support, which serves as physical barrier for wounds. However, its hydrophobicity can inhibit early cell attachment and induce inflammation in tissue. To improve the biocompatibility, we incorporated SF, a natural protein with low immunogenicity and extracellular matrix‑like structure. SF improves surface hydrophilicity and provides a favorable interface for cell proliferation. Moreover, combining SF with PLGA allows suitable sustained drug release. This mixture mitigates the initial burst release which is typical in hydrophilic systems and avoiding excessively slow diffusion in purely hydrophobic systems.

This strategy combines the advantages of both materials while minimizing their respective disadvantages. Both materials possess established safety records, supporting translational potential.

We have revised the Introduction section.

2. *The manuscript includes only DSC analysis for PLGA/SF and PLGA/SF/linagliptin, which is inadequate as a standalone technique. DSC reflects thermal behavior but cannot provide information on chemical structure, functional groups, or component ratios. It also cannot confirm characteristic interactions such as potential amide bond formation between PLGA and SF. To rigorously validate the material composition, the authors should incorporate additional characterization techniques (e.g., FTIR, Raman spectroscopy, XPS, or NMR) to verify functional groups, confirm the presence of all components, and determine their relative proportions.*

We fully appreciate the reviewer for the suggestions to incorporate additional characterization. We prepare the FTIR and XRD results of components and films (Figure R6A-B).

Pure PLGA displayed characteristic absorption peaks at 2999 cm⁻¹, 2949 cm⁻¹, 1747 cm⁻¹, 1455 cm⁻¹, 1386 cm⁻¹, 1171 cm⁻¹, 1083 cm⁻¹, 867 cm⁻¹, and 749 cm⁻¹, all peaks are consistent with the linear polymeric structure of PLGA. Similarly, pure silk fibroin (SF) showed peaks at 3286 cm⁻¹, 3071 cm⁻¹, 2936 cm⁻¹, 1640 cm⁻¹, 1513 cm⁻¹, 1229 cm⁻¹, and 1061 cm⁻¹, which align closely with reference spectra reported for SF.

In the PLGA/SF membranes, each observed peak could be clearly attributed to either PLGA or SF, with no significant shift or disappearance of the characteristic PLGA absorptions. This indicates that no pronounced chemical interaction occurred between the two components during fabrication.

Notably, in the PLGA/SF/Linagliptin membranes, we did not detect distinct FTIR peaks corresponding to linagliptin. This is likely due to its relatively low weight in the membranes and the overlap of its characteristic bands with the strong absorption signals from PLGA.

The results of XRD experiments are shown blow. Pure linagliptin exhibited characteristic diffraction peaks at multiple positions, including 2θ = 7.82°, 12.64°, 25.76°, 23.58°, 28.4°, and 21.28°. In contrast, no such distinct crystalline peaks were observed in the samples of the PLGA/SF and PLGA/SF/Linagliptin membranes. This absence indicates that the drug is homogeneously dispersed within the composite system in an amorphous state, which we attribute to its molecular-level integration during the electrospinning process.

These results are also updated in Figure 2 of the manuscript.

Figure R6. (A) The FTIR spectra of all components and membranes. (B) The XRD of Linagliptin and membranes.

3. *The figure caption for Figure 5 states: “(B) Immunofluorescence staining results for CD31 and α-SMA at 7 days and 14 days…”. However, the Day-14 CD31 and α-SMA staining images are missing from the presented data. The authors should include the missing Day-14 immunofluorescence results to maintain consistency.*

Thank you for pointing this out. We apologize for the typographical error. Since currently data is insufficient to support this claim, we have removed the text.

4. *The manuscript indicates that linagliptin is the primary therapeutic agent, with PLGA/SF functioning mainly as the delivery carrier. Therefore, the authors should additionally evaluate the carrier’s drug-loading efficiency, adsorption characteristics, and release kinetics. These parameters are essential for understanding the therapeutic mechanism and overall performance of the dressing.*

We appreciate the reviewer’s suggestion regarding the characterization of the drug delivery system. Understanding the release of drug-loaded materials is indeed critical. We have addressed these points as follows:

1. Drug-loading efficiency and release kinetics:

As requested, we have supplemented the manuscript with the drug-loading efficiency data, which indicates effective encapsulation of Linagliptin within the PLGA/SF membranes. The calculation formula of drug encapsulation efficiency is DEE (%) = Actual weight of Linagliptin in fiber/Theoretical weight of Linagliptin*100%, result showed that the DEE of our drug-loaded membrane is 82%. Furthermore, we have provided a detailed release kinetics of our materials in Figure 2A of our manuscript. To facilitate your review, we also display the results below (Figure R7).

Figure R7. Drug release curves of PLGA/SF/Linagliptin 1 and PLGA/SF/Linagliptin 2 wound matrix-induced membranes, measured over 0–15 days (PLGA/SF/Linagliptin 1: PLGA 1 g, SF 0.3 g, and linagliptin 6.5 mg; PLGA/SF/Linagliptin 2: PLGA 1 g, SF 0.3 g, and linagliptin 13 mg).

2. Adsorption characteristics

Regarding the "adsorption characteristics," we would like to clarify the fabrication mechanism employed in this study. The Linagliptin-loaded nanofibers were fabricated via electrospinning from a homogeneous solution, where the drug and polymers were co-dissolved. Unlike post-fabrication soaking methods where surface adsorption is the dominant mechanism, our strategy results in the physical encapsulation and molecular dispersion of the drug.

Instead, to thoroughly evaluate the characteristics of drug-carrier integration, we relied on FTIR and XRD analyses (presented in Figure R6).

We believe these data provide evidence for the carrier's drug-loading capability and the stability of the formulation.

5. *Because the treatment strategy involves applying the PLGA/SF/linagliptin electrospun membrane directly to the wound, the authors should provide data regarding its biodegradation behavior and long-term stability. These properties are crucial for assessing in vivo applicability, safety, and potential clinical translation.*

We agree with your valuable suggestions regarding the material's in vivo applicability, safety, and potential clinical translation. Linagliptin is a widely used and relatively safe drug in clinical practice. Since the wound dressing is designed as a non-implantable material, conducting long-term in vivo degradation experiments poses significant challenges; therefore, to explore its safety, we performed a long-term in vitro degradation test as an alternative to simulate the degradation curve (Figure R8A) and supplemented the hemolysis data to further validate biosafety (Figure R8B). Our results showed that in “DAY 28”, the weight of the membranes has dropped to about 15%, indicating that most of them has degraded. In clinical practice, wound dressing is changed once every three days, and most normal wounds heal within two weeks. So we think the degradation rate of the materials is moderate. And the extract material of both films did not show obvious hemolytic activity. Regarding in vivo tissue inflammation, our Myeloperoxidase (MPO) staining on Day 7 samples indicated that the drug-loaded materials supressed the inflammatory response (these data are exhibited in the Figure 5 of our manuscript).

These results indicate that the non-implanted material has a suitable degradation rate, does not cause severe inflammatory reactions or cause serious biosafety issues.

Figure R8.(A) The degradation curve of PLGA/SF and PLGA/SF/Linagliptin films. (B) The hemolysis test of the material extracts (EXT_1: the material extracts of PLGA/SF, EXT_2: the material extracts of PLGA/SF/Linagliptin).

6. *In Figure 4, two inconsistencies were noted: (i) at Day 0, the PLGA/SF/linagliptin group appears to include applied material, whereas no such material is visible in the PLGA/SF group; and (ii) the measuring ruler used at Day 7 differs from those used at other time points. The wound images should be standardized to ensure accuracy and fair comparison across groups.*

We are grateful for catching these details. For the first point, the PLGA/SF group actually did have the material applied. The issue was that the material is highly hydrophilic and became nearly transparent after absorbing wound fluid, which made it hard to see in the photo. We have replaced the image to fix this. Regarding the rulers, although they look different, the scale markings are exactly the same—I’ve included a comparison below to show you. We are sorry for ignoring this detail.

Figure R9. The comparision of different rules applied in animal experiments

Above all, we are very grateful for all the editors and reviewers, your suggestions are very enlightening and prompt us to carefully re-examine the research. We have benefited greatly from this process; these suggestions not only improved the quality of my paper but also helped develop our research.

Thank you and best regards

Sincerely,

Meng Yin

Shanghai Children's Medical Center, Shanghai Jiao Tong University School of Medicine, 1678 Dongfang Road, Shanghai 200127, People’s Republic of China;
